# Expression pattern and clinical value of Key RNA methylation modification regulators in ischemic stroke

**DOI:** 10.3389/fgene.2022.1009145

**Published:** 2022-10-03

**Authors:** Xinyue Zhang, Yuanlin Wang, Beibei Dong, Yi Jiang, Dan Liu, Keliang Xie, Yonghao Yu

**Affiliations:** ^1^ Department of Anesthesiology, Tianjin Medical University General Hospital, Tianjin, China; ^2^ Tianjin Institute of Anesthesiology, Tianjin, China; ^3^ School of Medicine, Nankai University, Tianjin, China; ^4^ Department of Critical Care Medicine, Tianjin Medical University General Hospital, Tianjin, China

**Keywords:** characteristic gene, epigenetics, immune infiltration, ischemic stroke, RNA methylation modification

## Abstract

Ischemic stroke (IS) is one of the major causes of death and disability worldwide, and effective diagnosis and treatment methods are lacking. RNA methylation, a common epigenetic modification, plays an important role in disease progression. However, little is known about the role of RNA methylation modification in the regulation of IS. The aim of this study was to investigate RNA methylation modification patterns and immune infiltration characteristics in IS through bioinformatics analysis. We downloaded gene expression profiles of control and IS model rat brain tissues from the Gene Expression Omnibus database. IS profiles were divided into two subtypes based on RNA methylation regulators, and functional enrichment analyses were conducted to determine the differentially expressed genes (DEGs) between the subtypes. Weighted gene co-expression network analysis was used to explore co-expression modules and genes based on DEGs. The IS clinical diagnosis model was successfully constructed and four IS characteristic genes (*GFAP*, *GPNMB*, *FKBP9*, and *CHMP5*) were identified, which were significantly upregulated in IS samples. Characteristic genes were verified by receiver operating characteristic curve and real-time quantitative PCR analyses. The correlation between characteristic genes and infiltrating immune cells was determined by correlation analysis. Furthermore, GPNMB was screened using the protein-protein interaction network, and its regulatory network and the potential therapeutic drug chloroquine were predicted. Our finding describes the expression pattern and clinical value of key RNA methylation modification regulators in IS and novel diagnostic and therapeutic targets of IS from a new perspective.

## Introduction

Ischemic stroke (IS) is a serious cerebrovascular disease characterized by a high disability rate and mortality, imposing a massive burden on society (Zhou et al., 2019). Current evidence shows that the focus of IS treatment is emergency intervention and long-term secondary prevention (Herpich and Rincon, 2020). However, owing to the narrow therapeutic window and hemorrhage-related complications, the clinical treatment options for IS are very limited and only a minority of patients benefit (Henderson et al., 2018). Therefore, effective diagnostic biomarkers and treatments are urgently needed to improve early diagnosis, reduce mortality, and improve prognosis of IS.

Several studies have been performed to improve the understanding of the molecular mechanisms of IS based on microarray and bioinformatics analysis. A previous study isolated 10 hub genes and five key miRNAs between IS and normal control groups by analyzing two datasets (GSE58294 and GSE16561) (Yang et al., 2022). Li et al. (2020) studied IS from the perspective of immune regulation and identified immune-related gene expression modules and hub genes in the peripheral blood of patients with IS, which might become important targets for immunotherapy of IS. However, these studies merely identified differentially expressed genes (DEGs), without exploring the detailed molecular mechanisms and potential drug molecules.

As a research hotspot in recent years, the post-transcriptional chemical modification of RNA is rapidly emerging as a pivotal player in regulating gene expression. To date, more than 170 types of RNA modifications have been identified that modify coding and noncoding RNAs, which account for more than 50% of methylations (Boccaletto et al., 2022). RNA methylation, an abundant and widely studied epigenetic modification, plays an important role in modulating multiple biological functions (Zhou et al., 2020). The occurrence of RNA methylation is reversible and dynamically regulated by groups of proteins called RNA-modifying proteins, including “writers” (methyltransferases), “erasers” (demethylases), and “readers” (methyl binding proteins) (Zaccara et al., 2019). N1-methyladenosine (m1A), N6-methyladenosine (m6A), and 5-methylcytosine (m5C) are common types of eukaryotic RNA methylation modifications (Xu et al., 2021), among which m6A RNA methylation has been reported to be highly enriched in the mammalian brain and closely associated with the pathological mechanism of IS (Yu et al., 2021). For instance, Xu S. et al. (2020) found that lnc-D63785 m6A methylation leads to the accumulation of miR-422a and neuronal death in an oxygen-glucose deprivation/reperfusion model. Moreover, as one of the m6A “readers,” YTHDC1 has been found to alleviate brain injury through the PTEN/Akt pathway and provide a potential therapeutic target for treating IS (Zhang Z. et al., 2020). However, as new types of RNA methylation, the relationship between m1A- and m5C-related regulators and IS has not been reported, and their mechanisms need to be further explored.

A growing body of research has confirmed that the immune microenvironment plays a vital role in IS (Zera and Buckwalter, 2020; Liu et al., 2021). Following IS, peripheral immune cells migrate through the broken blood-brain barrier to the damaged area and activate host immune cells, such as microglia (Chavda et al., 2021). Infiltrated inflammatory cells and the activated immune response lead to the dysfunction of the immune microenvironment, which dramatically hinders neurological functional recovery (Shi et al., 2022). Further evidence indicates that RNA methylation modifications are involved in immune regulation, especially in the tumor immune microenvironment. For example, m5C regulators have been shown to promote the expression and infiltration of CD8^+^ T cells and are associated with poor prognosis in patients with lung squamous cell carcinoma (Pan et al., 2021). The m6A-binding protein YTHDF1 facilitates tumor immune escape by impairing the cross-presentation of tumor neoantigens and cross-priming of CD8^+^ T cells (Han et al., 2019). However, the role of RNA methylation regulators in immune infiltration in IS have yet to be explored.

In this study, we first comprehensively analyzed the GSE97537 dataset to identify differentially expressed RNA methylation-related regulators (m1A, m6A, and m5C) and evaluate immunocyte infiltration in IS and control samples, and we identified IS-related subtypes. Gene ontology (GO) and Kyoto Encyclopedia of Genes and Genomes (KEGG) pathway enrichment analyses were performed to identify DEGs between subtypes. Weighted gene co-expression network analysis (WGCNA) was used to identify the co-expressed genes and modules. Then, we constructed a clinical diagnostic model of IS and identified characteristic genes. Protein-protein interaction (PPI) networks, transcription factor (TF) correlation, competing endogenous RNA (ceRNA) networks, and potential drug molecules for IS therapy were identified based on characteristic genes. Our study may provide insight into the role of RNA methylation in pathogenesis and immune infiltration in IS.

## Materials and methods

### Data acquisition and preprocessing

Gene expression profile data, focusing on ischemic reperfusion, were obtained from the Gene Expression Omnibus database (https://www.ncbi.nlm.nih.gov/geo/). In total, 22 samples from GSE97537 (7 IS and five control rat samples) (Wang et al., 2015) and GSE61616 (5 IS and five control rat samples) were selected. The same platform, GPL1355, was used for the two datasets. Detailed information from GSE97537 and GSE61616 is listed in Supplementary Table S1. RNA methylation-related regulators from previous studies, including 11 m1A methylation regulators (Gao et al., 2021), 21 m5C methylation regulators (Chen et al., 2020), and 23 m6A methylation regulators (Zhao et al., 2017; He et al., 2019) were collected. The “Affy” package (Ritchie et al., 2015) was utilized to normalize gene expression values from the two datasets. Nextly Log2 transformation was also carried out. Principal component analysis (PCA) was performed to detect the distribution of samples in the two groups. GSE97537 was considered the primary analysis dataset and training set. GSE61616 was chosen as the testing set to check the diagnostic ability of this diagnosis model.

### Screen of RNA methylation regulators

The differential expression analysis and visualization of m1A, m5C, and m6A methylation regulators between IS samples and control samples were performed using the “limma” and “pheatmap” packages. The “RCircos” package (Zhang et al., 2013) in R, which can display the chromosomal location of DEGs, was used. Next, the correlation and interaction between DEGs of m1A, m5C, and m6A methylation regulators were calculated based on the Pearson algorithm. Gene interaction networks showing these factors were drawn using the “Corrplot” package.

### Infiltration characteristics of the immune microenvironment in IS

CIBERSORTx (https://cibersort.stanford.edu/), an R tool for the deconvolution of expression matrices of immune cell subtypes, was designed by combining linear support vector regression and immune infiltration theory (Chen et al., 2018). We used the CIBERSORTx algorithm to profile the landscape of 22 types of immune cells in the immune microenvironments of the IS and normal groups according to gene expression levels in datasets. The correlation among immune cells was considered very influential to understand immune pathway and function. Therefore, the correlation coefficients between immune cells were calculated by Spearman analysis and visualized through heatmaps. Statistical differences in the proportion of infiltrating immunocytes between IS and control groups were calculated by Wilcoxon test using R software (v. 3.5.1).

### Identification of IS-related molecular subtype

Consensus clustering was performed using the “ConsensusClusterPlus” package (Wilkerson and Hayes, 2010) to identify IS subgroups based on differentially expressed RNA methylation regulators. By combining consensus cumulative distribution function (CDF) plots, delta area plots, tracking plots, and clustering heatmaps, the optimal number of clusters was identified. These clusters were defined as IS-related molecular subtypes.

### GO and KEGG enrichment analyses

The “limma” package was used to screen DEGs between the subtypes. The cut-off criteria for statistical significance were adjusted *p* value (*P*_adj_) < 0.05 and logFC >1. GO analysis (Ashburner et al., 2000) is a major bioinformatics tool designed for complex functional enrichment analyses, composed of annotations of biological process (BP), molecular function (MF), and cellular component (CC). KEGG (Kanehisa and Goto, 2000), an integrated database resource, is used to understand high-level functions and utilities of biological systems from genomic and molecular-level information. GO annotation and KEGG pathway enrichment analyses of DEGs were performed using the “clusterProfiler” package (Yu et al., 2012). Results with a false discovery rate <0.05 were considered statistically significant. The pathway with the highest enrichment of DEGs in KEGG analysis was visualized using the “Pathview” package (Luo and Brouwer, 2013).

### Gene set enrichment analysis (GSEA) and gene set variation analysis (GSVA)

GSEA, an analytical method based on the entire gene expression matrix, was conducted to derive the significant differences in biological processes between the IS subtypes. Reference gene sets, “c2. all.v7.5.2. entrez.gmt,” were downloaded from the Molecular Signature Database (Liberzon et al., 2015). *P*_adj_ < 0.05 and |normalized enrichment score| > 1 were considered to indicate statistical significance. GSVA, a nonparametric unsupervised analysis method, was used to evaluate different pathways enriched in the different samples. In our study, GSVA was performed using the “GSVA” package (Hänzelmann et al., 2013).

### WGCNA

WGCNA is a systems biology method that can be used to identify modules of highly correlated genes among different samples and identify candidate biomarkers or potential therapeutic targets based on the association of modules to one another and to phenotype (Yue et al., 2016). The top 1,000 genes in gene expression data of IS samples, which were ranked by median absolute deviation (MAD), were analyzed using the “WGCNA” package (Langfelder and Horvath, 2008). Then, we removed outliers and set an optimal soft threshold. The settings of minModuleSize = 25 and set height = 0.15 were used to obtain the final co-expression modules. Finally, genes in the most important modules were screened.

### Construction of a diagnostic model

To identify characteristic genes associated with IS and analyze their diagnostic ability, least absolute shrinkage and selection operator (LASSO) regression was performed. A diagnostic model was constructed based on the training dataset, GSE97537. Further validation of this model was performed on the GSE61616 dataset. Receiver operating characteristic (ROC) curves drawn using the “ROCR” package (Sing et al., 2005) were used to illustrate the diagnostic ability of this model on the test set.

### Correlation analysis between characteristic genes and immune cell infiltration

The expression levels of characteristic genes associated with IS and immune cell infiltration score were integrated. Spearman correlation analysis was used to determine the correlation between characteristic genes and immunocyte fractions. Detailed results were displayed as a lollipop plot.

### Establishment of an animal model and RT-qPCR

All experimental procedures were approved by the Animal Experimental Ethics Committee of Tianjin Medical University General Hospital. Male C57BL/6J mice (aged 6–8 weeks, 20–25 g) were used to establish the middle cerebral artery occlusion (MCAO) animal model. Specific operations and evaluation methods are detailed in a previous study (Espinosa et al., 2020). Sham-operated mice underwent the same surgical procedures except for the occlusion of the middle cerebral artery. Twenty-4 hours after reperfusion, mice were sacrificed by euthanasia and the cerebral cortex of the lesioned side was removed from the mice. Total RNA was extracted from the cortex using TRIzol reagent (Invitrogen, Carlsbad, CA, United States ) and used as a template for reverse transcription into cDNA using a cDNA synthesis kit (Thermo Scientific, Waltham, MA, United States ). Then, RT-qPCR amplification was carried out. *GAPDH* was used for normalization. Primer sequences are shown in Supplementary Table S2.

### Construction of protein-protein interaction networks and hub gene regulatory networks

PPI networks were constructed in the Search Tool for the Retrieval of Interacting Genes (STRING) online database (http://string-db.org; v. 10.5). Visualization was performed in Cytoscape (v. 3.9.0) (Shannon et al., 2003). Maximal clique centrality (MCC) was calculated using CytoHubba (Chin et al., 2014), a Cytoscape plugin. Genes with the highest MCC value were selected as hub genes.

### Prediction of ceRNA network

The ENCODE database (https://www.encodeproject.org/) (Davis et al., 2018) was used to screen possible TFs of the hub gene. To explore the potential relationship between the hub gene and various noncoding RNAs, we constructed a ceRNA network using Cytoscape. The interaction information between mRNA and miRNA and between miRNA and lncRNA were predicted using miRTarBase (Huang et al., 2022) and StarBase database (Li et al., 2014), respectively.

### Construction of drug-gene network and molecular docking

We used the Comparative Toxicogenomics Database (Davis et al., 2021) (http://ctdbase.org/) to predict drug molecules that might be useful in the treatment of IS by targeting hub genes. Cytoscape was used to visualize the interaction network between hub genes and drug molecules. According to the targeting relationship and reference scores of these potential components, a potential therapeutic drug was identified. PubChem (https://pubchem.ncbi.nlm.nih.gov/) and PDB (http://www.rcsb.org/) databases (Burley et al., 2017), which contain three-dimensional structures of small molecules and large-sized proteins, were searched and detailed structures for drug and hub mRNA and proteins were obtained. Autodock (v. 4.2.6) and Pymol (v. 2.3.0) were used to calculate and visualize the results of docking for drugs and mRNA/proteins. To verify docking results, YASARA was utilized through another algorithm (Krieger and Vriend, 2014). I Mutant3.0 was also utilized to identify function of important binding location.

### Statistical analysis

All data processing and analyses were completed in R software (v. 4.1.1). Statistical significance between non-normally distributed variables was analyzed using the Mann-Whitney U test (Wilcoxon rank sum test). Correlation coefficients among different genes were calculated using Pearson correlation analysis. Spearman correlation analysis was used to calculate the correlation coefficients between different immune cells and with genes. *p* < 0.05 was considered to indicate statistically significant results.

## Results

### Analysis flow chart and data preprocessing

**FIGURE 1:**
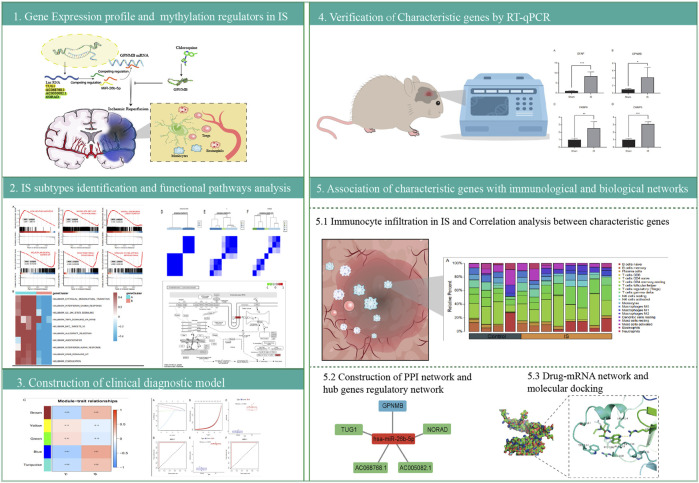
Protocol flowchart.

The analysis flow chart of this study is shown in Figure 1. Gene expression data of the two datasets were normalized to eliminate the batch effect (Supplementary Figures S1A, B). According to the results of PCA, IS samples and control samples were well-classified (Supplementary Figures S1C, D).

### Expression profile and chromosomal localization of m1A, m5C, and m6A regulators in IS.

**FIGURE 2:**
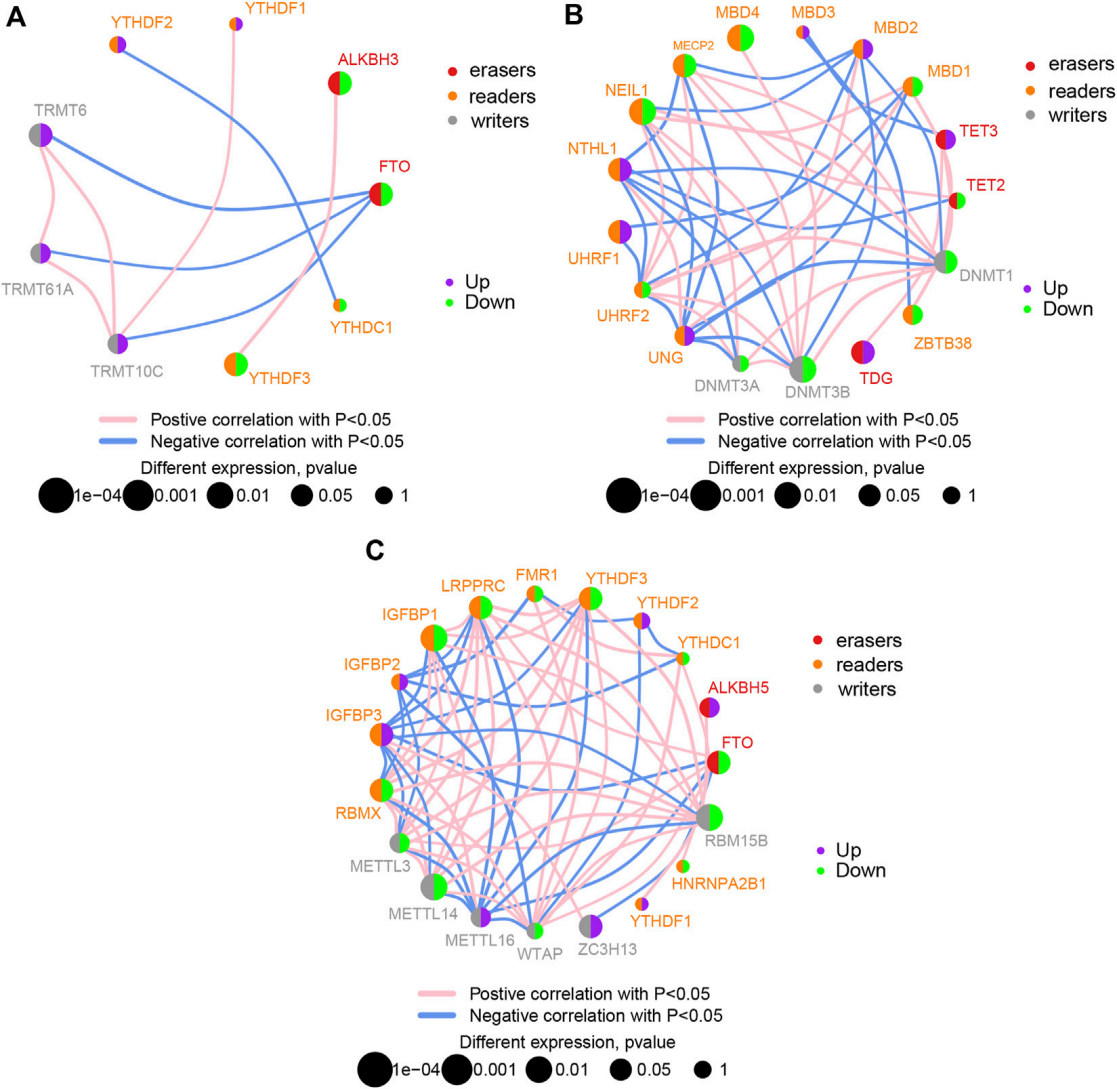
Correlation between differentially expressed genes. Correlation network diagram of differentially expressed m1A regulators **(A)**, m5C regulators **(B)**, and m6A regulators **(C)** in ischemic stroke samples.

DEGs of m1A, m5C, and m6A regulators (readers, writers, and erasers), with significant differences, were clearly separated in the heatmap according to groups (Supplementary Figure S2A). Detailed chromosomal locations of DEGs (6 m1A regulators, 15 m5C regulators, and 13 m6A regulators) were displayed using a chromosomal circle diagram (Supplementary Figure S2B–D). m1A-related genes were mainly located on chromosomes 1, 3, 8, 11, and 14; m5C-related genes were mainly located on chromosomes 2, 3, 19, and 18; and m6A-related genes were mainly located on chromosomes X, 17, and 7. The correlation and interaction among DEGs are exhibited in Figures 2A–C. DEGs belonging to m1A, m5A, and m6A could be linked in each network.

### Immunocyte infiltration and correlation in IS and control samples

**FIGURE 3:**
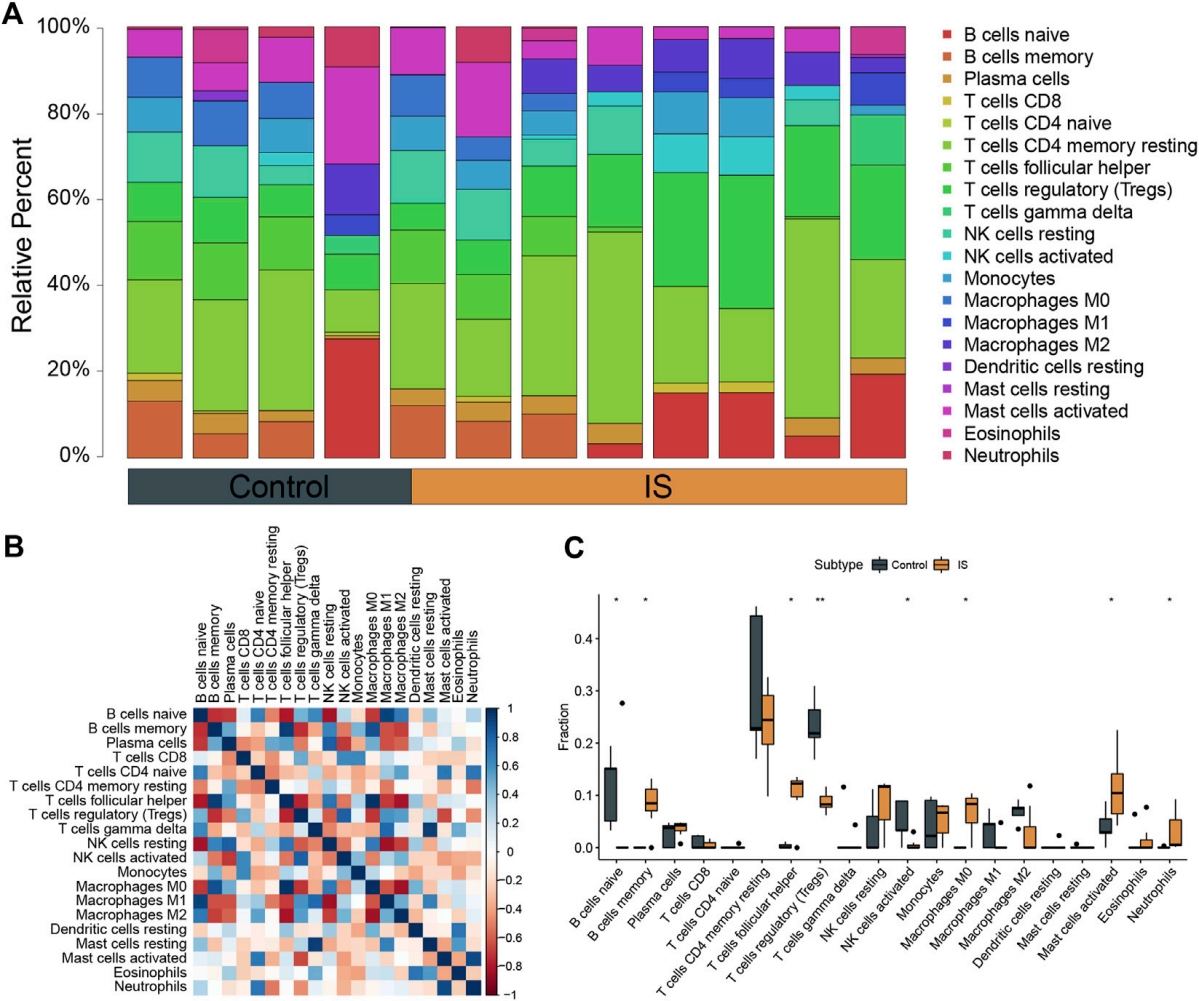
Analysis of immune infiltration in ischemic stroke (IS). **(A)** Overall expression of 20 infiltrating immune cell types in IS and control groups. **(B)** Correlation heat map of immune infiltrating cells. **(C)** Differential expression of infiltrating immune cells between the groups.

The CIBERSORTx algorithm was used to calculate the abundance ratios of 22 types of immune cells, and cells with abundance ratios of 0 were removed in the subsequent analysis (Figure 3A). The correlation coefficients between immune cells were analyzed. Positive relationships were observed between M0 macrophages and follicular helper T cells and between gamma delta T cells and resting mast cells. Negative relationships were observed between regulatory T cells and follicular helper T cells, M1 macrophages and resting NK cells, and M2 macrophages and M0 macrophages (Figure 3B). Significant differences in the proportion of immune cells between IS and control samples were calculated. Differences in naïve B cells, memory B cells, follicular helper T cells, activated NK cells, M0 macrophages, activated mast cells, neutrophils (*p* < 0.05), and T cells regulatory (Tregs) (*p* < 0.01) between IS and control samples were significant (Figure 3C).

### Identification of IS subtypes based on the RNA methylation-related regulators

Gene expression profiles of 34 differentially expressed RNA methylation-related regulators were constructed to investigate the IS molecular subtypes. The CDF plots, delta area plots, and tracking plot Supplementary Figure S3A–C) were used to assess the appearance of different *k* values. Three kinds of selection on *k* (*k =* 2, 3, 4) and the probable separating subtypes in ConsensusClusterPlus are separately shown in Supplementary Figure S3D–F. The optimal division was reached when *k* = 2, thus, two IS subtypes were identified.

### GO functional enrichment analysis and KEGG pathway analysis

**FIGURE 4:**
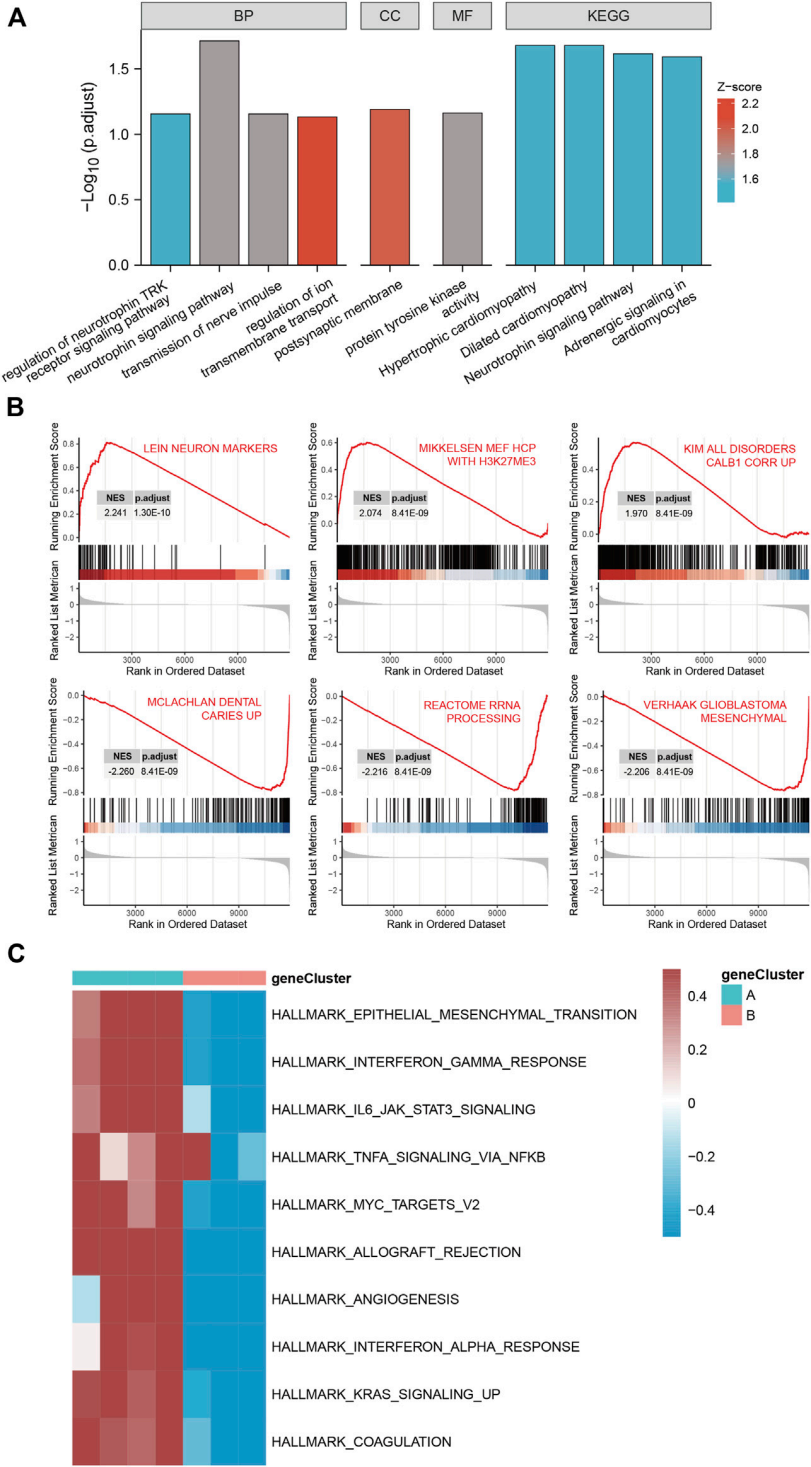
Gene ontology (GO), Kyoto Encyclopedia of Genes and Genomes (KEGG), gene set enrichment analysis (GSEA) and gene set variation analysis (GSVA) of DEGs between two IS subtypes. **(A)** Items with minimum Padj of GO enrichment analysis (biological processes, cellular components, and molecular functions) and KEGG pathway enrichment. **(B)** Clustering of the top three pathways with the highest and lowest normalized enrichment scores in GSEA. **(C)** Heat map of the top 10 enrichment results with the highest median absolute deviation of GSVA; red represents upregulation and blue represents downregulation.

GO functional enrichment analysis and KEGG pathway analysis were performed on 28 DEGs between the IS subtypes to obtain more detailed information on their potential functions and correlated pathways. According to the results of GO functional enrichment analysis, the DEGs were mainly enriched in BP: regulation of neurotrophin TRK receptor signaling pathway, neurotrophin signaling pathway, transmission of nerve impulse, and regulation of ion transmembrane transport; in CC: postsynaptic membrane; and in MF: protein tyrosine kinase activity (Figure 4A). Hypertrophic cardiomyopathy, selected as the pathway with the highest score in KEGG analysis, is shown in Supplementary Figure S4. The overall results of GO and KEGG analysis are shown in Table 1, 2.

**TABLE 1:** GO enrichment analysis.

GO Enrichment Results								
**Category**	**ID**	**Description**	**BgRatio**	**pvalue**	**p.adjust**	**qvalue**	**geneID**	**Count**
BP	GO:0038179	neurotrophin signaling pathway	43/17859	0.000022	0.02064	0.015211	Wasf1/Ntrk3/Agt	3
BP	GO:0051386	regulation of neurotrophin TRK receptor signaling pathway	16/17859	0.000188	0.076186	0.056146	Wasf1/Agt	2
BP	GO:0019226	transmission of nerve impulse	96/17859	0.000247	0.076186	0.056146	Cacng3/Ntrk3/Agt	3
BP	GO:0048011	neurotrophin TRK receptor signaling pathway	31/17859	0.000721	0.157632	0.116168	Wasf1/Agt	2
CC	GO:0045211	postsynaptic membrane	322/18211	0.006574	0.158162	0.12973	Cacng3/Ntrk3/Lzts1	3
CC	GO:0043235	receptor complex	395/18211	0.011483	0.158162	0.12973	Cacng3/Ntrk3/Tyro3	3
CC	GO:0014069	postsynaptic density	425/18211	0.013980	0.158162	0.12973	Cacng3/Rnf112/Lzts1	3
CC	GO:0031209	SCAR complex	12/18211	0.014405	0.158162	0.12973	Wasf1	1
MF	GO:0004714	transmembrane receptor protein tyrosine kinase activity	106/16532	0.000361	0.020574	0.017646	Ntrk3/Tyro3/Matk	3
MF	GO:0019199	transmembrane receptor protein kinase activity	123/16532	0.000558	0.020574	0.017646	Ntrk3/Tyro3/Matk	3
MF	GO:0004713	protein tyrosine kinase activity	124/16532	0.000571	0.020574	0.017646	Ntrk3/Tyro3/Matk	3
MF	GO:0016247	channel regulator activity	145/16532	0.000901	0.021156	0.018146	Cacng3/Fxyd7/Agt	3

**TABLE 2:** KEGG enrichment analysis.

KEGG Enrichment Results							
**ID**	**Description**	**BgRatio**	**pvalue**	**p.adjust**	**qvalue**	**geneID**	**Count**
rno05410	Hypertrophic cardiomyopathy	91/8947	0.0014946	0.023111	0.01509998	Cacng3/Agt	2
rno05414	Dilated cardiomyopathy	94/8947	0.0015939	0.023111	0.01509998	Cacng3/Agt	2
rno04722	Neurotrophin signaling pathway	120/8947	0.0025834	0.024973	0.01631621	Ntrk3/Matk	2
rno04261	Adrenergic signaling in cardiomyocytes	148/8947	0.003903	0.028296	0.01848773	Cacng3/Agt	2

### GSEA and GSVA

The top three pathways with the highest and lowest normalized enrichment score are shown in Figure 4B (highest: LEIN_NEURON_MARKERS, MIKKELSEN_MEF_HCP_WITH_H3K27ME3, and KIM_ALL_DISORDERS_CALB1_CORR_UP; lowest: MCLACHLAN_DENTAL_CARIES_UP, REACTOME_RRNA_PROCESSING, and VERHAAK_GLIOBLASTOMA_MESENCHYMAL). The overall results of GSEA are shown in Table 3. The top 10 enrichment results in GSVA with the highest MAD are shown in Figure 4C.

**TABLE 3:** GSEA analysis results.

GSEA Analysis Results							
ID	setSize	enrichmentScore	NES	pvalue	p.adjust	qvalues	rank
LEIN_NEURON_MARKERS	59	0.814076967	2.241206	1.30E-10	1.08E-08	7.07E-09	1,584
MIKKELSEN_MEF_HCP_WITH_H3K27ME3	440	0.601882423	2.074171	1.00E-10	8.41E-09	5.53E-09	1,670
KIM_ALL_DISORDERS_CALB1_CORR_UP	468	0.567695316	1.970307	1.00E-10	8.41E-09	5.53E-09	1968
WP_SYNAPTIC_VESICLE_PATHWAY	47	0.744183724	1.96983	1.02E-05	0.000171	0.00011	1869
REACTOME_ION_HOMEOSTASIS	52	0.736104382	1.947812	5.60E-06	0.000108	7.07E-05	1727
POOLA_INVASIVE_BREAST_CANCER_UP	194	-0.739888555	-2.17464	1.00E-10	8.41E-09	5.53E-09	1744
REACTOME_INTERLEUKIN_10_SIGNALING	42	-0.903693749	-2.19738	1.00E-10	8.41E-09	5.53E-09	516
VERHAAK_GLIOBLASTOMA_MESENCHYMAL	169	-0.76491492	-2.20585	1.00E-10	8.41E-09	5.53E-09	1,481
REACTOME_RRNA_PROCESSING	135	-0.785930466	-2.21597	1.00E-10	8.41E-09	5.53E-09	1958
MCLACHLAN_DENTAL_CARIES_UP	169	-0.783539634	-2.25956	1.00E-10	8.41E-09	5.53E-09	1,083

### WGCNA

**FIGURE 5:**
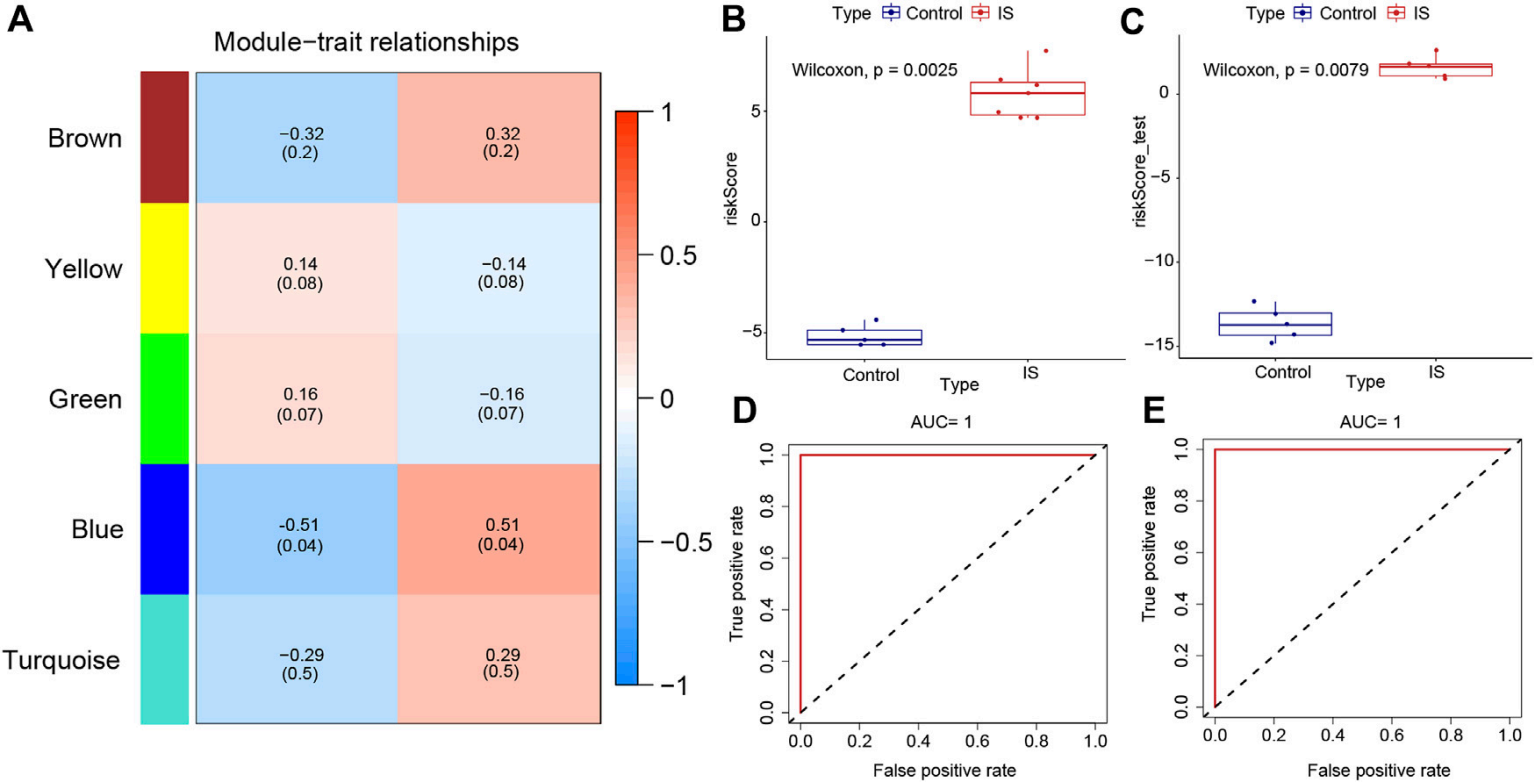
Weighted gene co-expression network analysis and the construction of diagnostic model. **(A)** Heat map of correlations between modules and samples. **(B)** Differences in model scores between ischemic stroke (IS) and control groups in training set. **(C)** Differences in model scores between IS and control groups in the testing set. **(D)** Corresponding receiver operating characteristic (ROC) curves and area under the curve (AUC) values in the training set. **(E)** Corresponding ROC curves and AUC values in testing set.

The “WGCNA” package in RStudio was used to identify co-expressed genes and modules. The clustering results based on characters showed good clustering, with no outlier samples were detected (Supplementary Figure S5A). In total, five modules were identified in WGCNA (Supplementary Figure S5B). By comparing the correlation between module genes and the two IS subtypes, blue modules with the largest correlation difference were identified as the most important modules (Figure 5A). DEGs in blue modules were subsequently analyzed.

### Construction of clinical diagnostic model

Datasets GSE97537 and GSE61616 were regarded as the training and testing sets, respectively. With the increase of parameter λ, the selected characteristic parameters decreased and absolute value of coefficients increased (Supplementary Figure S5C). After the simulation and selection of characteristic parameters, two models were obtained (optimal model and minimalist model) (Supplementary Figure S5D). We selected the minimalist model to construct the diagnostic model and identified four genes as characteristic genes of IS, namely, *GFAP*, *GPNMB*, *FKBP9*, and *CHMP5*. Then, model scores of IS and control groups in the two datasets were analyzed, and the results showed significant differences between two groups (Wilcoxon test, *p* < 0.05) (Figures 5B,C). The ROC curves of the training and testing sets were plotted to determine the area under the curve (AUC) value to verify the accuracy of the diagnostic model. The AUC values were 1 and 0.748, respectively (Figures 5D,E).

### Correlation analysis between characteristic genes and immune cell infiltration

**FIGURE 6:**
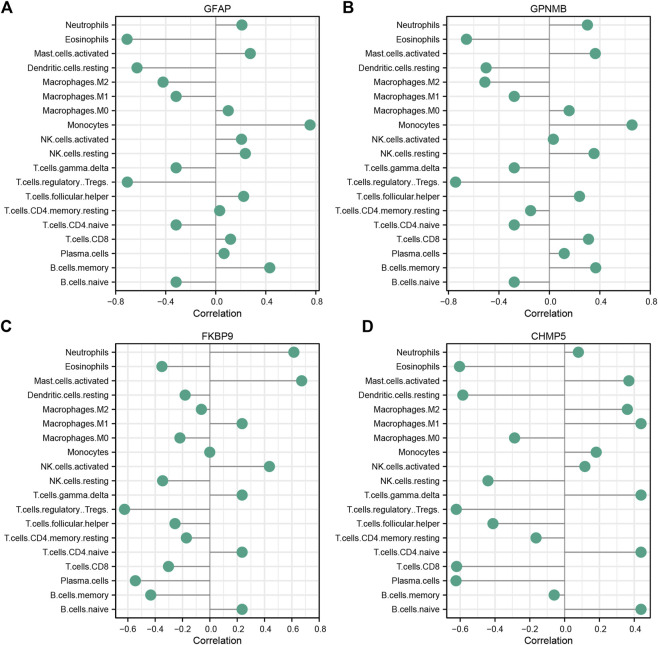
Correlation between characteristic ischemic stroke genes GFAP **(A)**, GPNMB **(B)**, FKBP9 **(C)**, and CHMP5 **(D)** and infiltrating immune cells.

The correlation of characteristic genes and immunocyte fractions was determined by Spearman correlation analysis and the results are displayed as a lollipop plot (Figures 6A–D).

### Characteristic genes were verified by RT-qPCR

**FIGURE 7:**
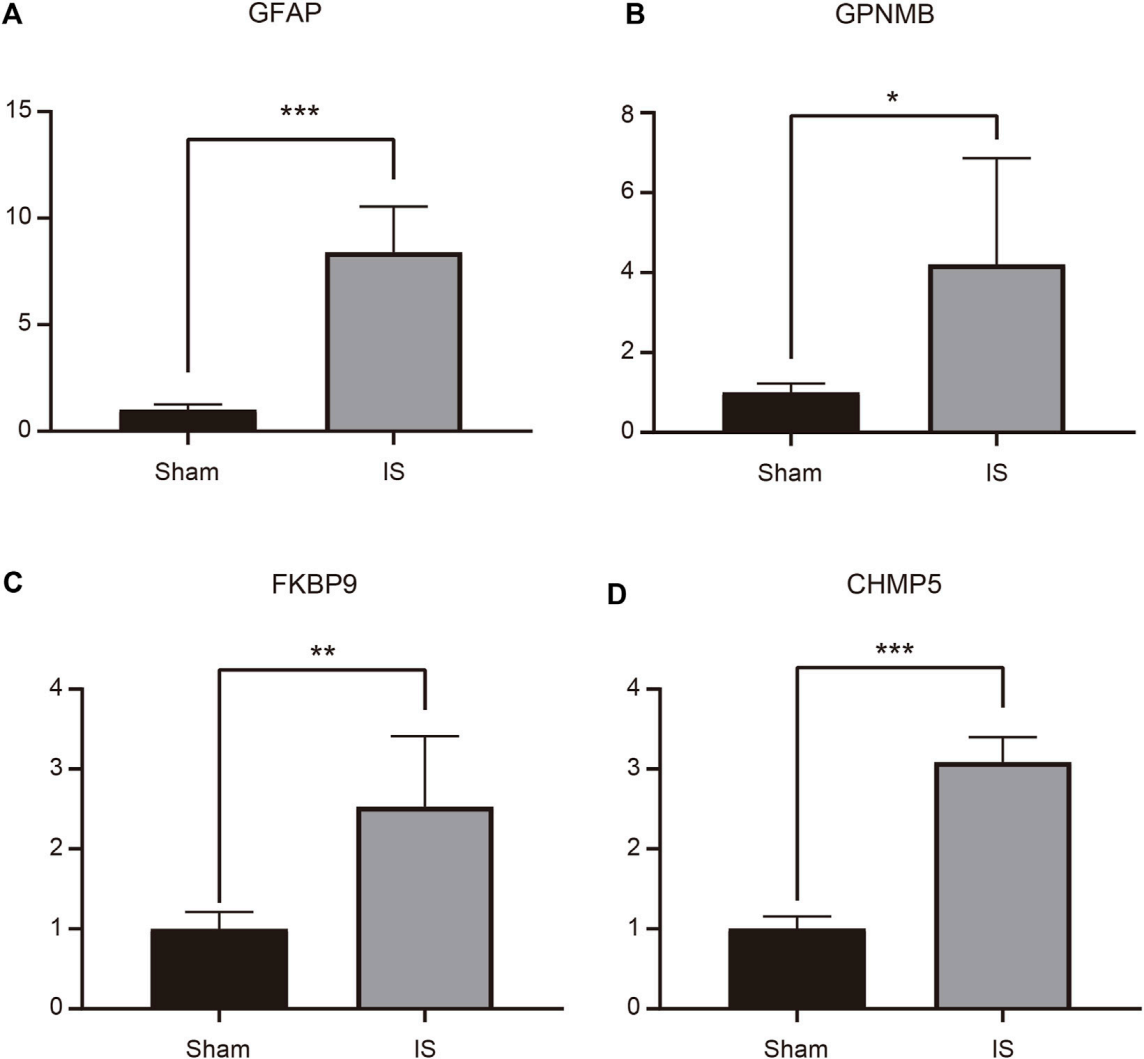
Expression of GFAP **(A)**, GPNMB **(B)**, FKBP9 **(C)**, and CHMP5 **(D)** in ischemic stroke brain tissue determined by RT-qPCR. *p < 0.05, **p < 0.01, ***p < 0.001.

An MCAO mouse model was established to simulate IS, and RT-qPCR was performed to verify the expression of the four characteristic genes in the cerebral cortex of MCAO and Sham-operated mice. The expression of *GFAP*, *GPNMB*, *FKBP9*, and *CHMP5* was significantly higher in MCAO than in Sham-operated samples (*p* < 0.05) (Figure 7).

### Construction of PPI network and hub gene regulatory network

**FIGURE 8:**
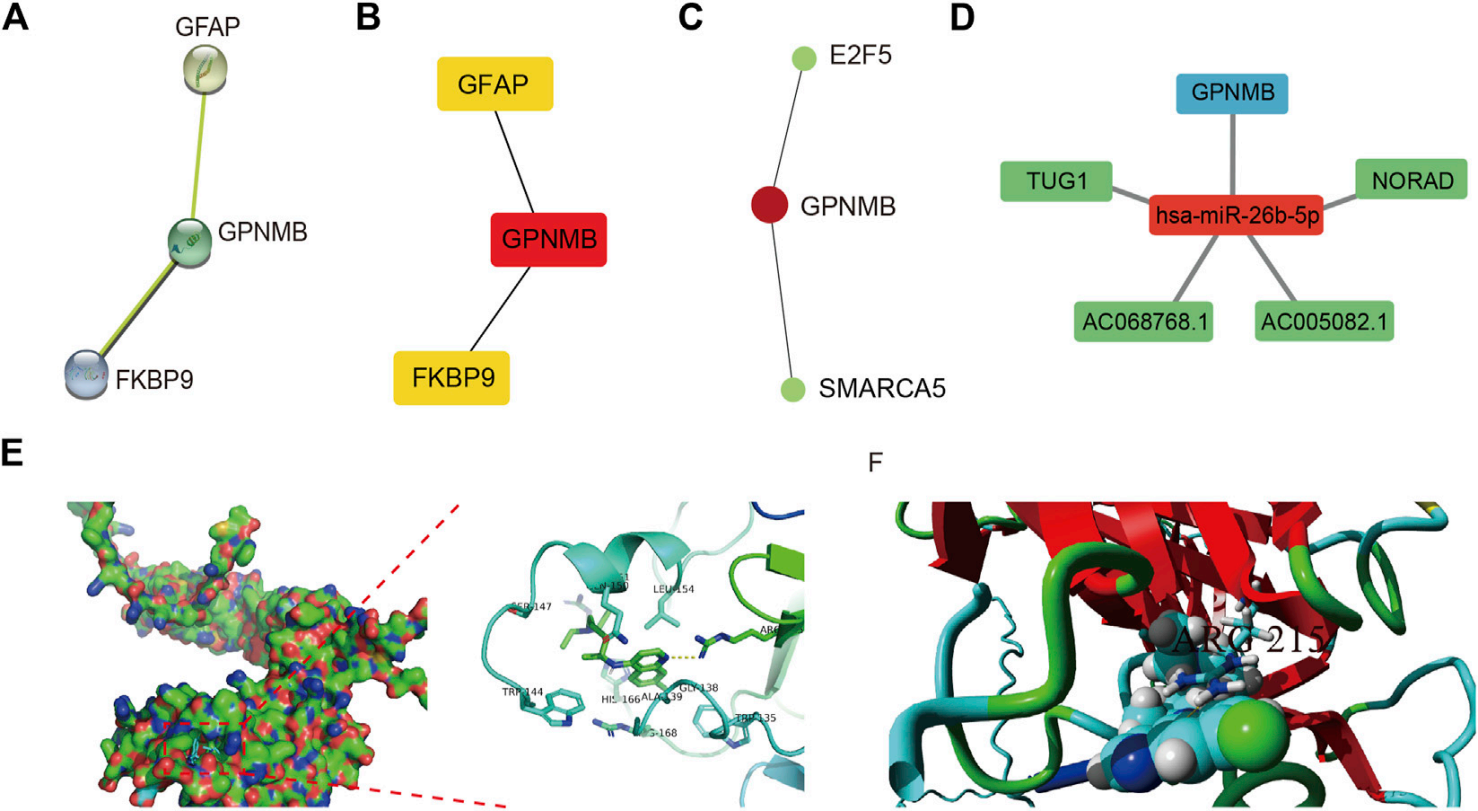
Protein-protein interaction (PPI) network, transcription factor (TF) correlation network, competing endogenous RNA (ceRNA) network and drug-molecular docking of the hub gene. **(A)** PPI network; each node represents a different gene. **(B)** Maximal clique centrality (MCC) value of each gene was calculated. The deeper the color the higher, the MCC value of genes. **(C)** TF correlation network of the hub gene. Red represents genes and green represents TFs. **(D)** ceRNA network of the hub gene. Red represents miRNA, blue represents mRNA, and green represents lncRNA. **(E)** Docking results between GPNMB and chloroquine in Autodock. Hydrogen bond between two was marked in yellow dotted line. **(F)** Docking results of YASARA. Binds marked in yellow represent hydrogen bind.

The STRING database was used to construct the PPI network of characteristic genes associated with **IS** (Figure 8A). The interactions between genes were imported into Cytoscape, and the MCC value of each gene was calculated by CytoHubba. *GPNMB*, which had the highest MCC value, was identified as a hub gene (Figure 8B). The interaction network between *GPNMB* and TFs was obtained using the ENCODE database (Figure 8C). An miRNA, has-miR-26b-5p, was predicted to interact with *GPNMB*, and lncRNAs related to has-miR-26b-5p were further predicted. Subsequently, the ceRNA network of *GPNMB* was constructed based on these prediction results (Figure 8D).

### Drug-mRNA network and molecular docking

In total, 241 drug molecules were predicted as potential drugs targeting GPNMB (Supplementary Figure S6). Putative GPNMB-drug interaction networks are shown in Figure 8E. The binding energy between chloroquine and GPNMB in most possible mode, of the lowest binding energy, was -5.6 kcal/mol, less than -5 kcal/mol, indicating small molecules were capable of binding with protein receptor to an extent. A hydrogen bond existed between alanine in GPNMB and small drug molecules with a distance of 3.2 Å. This specific bond was observed as basic interaction in the docking pocket area. According to results of YASARA software, the most possible docking mode was shown in (Figure 8F). Binding location between two algorithms were the same, both of hydrogen bond with ARG215. I-Mutant 3.0 web server, assessing the influence of one amino acid dot mutation on GPNMB protein through free energy stability change (DDG) was utilized for ARG 215 (Fang et al., 2019). The average DDG was -0.73 kcal/mol. According to reference criteria, DDG < -0.5, indicating large decrease of protein structure stability (Lim et al., 2021). In our article, ARG 215 was hydrogen binding location, results of mutation revealing the important function of this location.

## Discussion

Stroke, as a sudden disorder of cerebral blood circulation, has emerged as the second leading cause of death and disability worldwide owing to the lack of early diagnosis and effective treatment. IS the most prevalent form of stroke, accounting for approximately 75–80% of cases (Krishnamurthi et al., 2018). Increasing evidence supports the involvement of epigenetic alterations in the pathogenesis of IS (Stanzione et al., 2020). However, as one of the most ubiquitous epigenetic modifications in mammalian cells, the role of RNA methylation modification in the regulation of IS remains unclear. To determine the role of RNA methylation-related regulators (m1A, m6A, and m5C) in the pathogenesis and immune microenvironment of IS, we determined the overall expression of RNA methylation regulators and immune infiltration in rat IS gene sets and divided IS into two molecular subtypes according to the expression levels of RNA methylation regulators. An IS clinical diagnosis model was successfully constructed and four upregulated characteristic genes were identified, which were significantly negatively correlated with the degree of Treg infiltration. Furthermore, we identified a hub gene by PPI network analysis and predicted its regulatory networks and the potential therapeutic drug, chloroquine.

Two IS molecular subtypes were constructed based on the differentially expressed RNA methylation-related regulators between IS and normal samples, and 28 DEGs were identified between them. GO analysis revealed that these DEGs were engaged in BP of regulation of neurotrophin TRK receptor signaling pathway, neurotrophin signaling pathway, transmission of nerve impulse, and regulation of ion transmembrane transport, which have been referred to as the main mechanisms of IS (Li et al., 2021; Zhu et al., 2022). Additionally, the protein products of these DEGs are mainly distributed in the postsynaptic membrane and primarily involved in regulating protein tyrosine kinase activity. A previous study has revealed that protein tyrosine kinase activity, which is closely associated with various synaptic and cellular functions in the brain, is upregulated in IS (Takagi, 2014). Pathway enrichment assessments determined that the top four enriched pathways were hypertrophic cardiomyopathy, dilated cardiomyopathy, neurotrophin signaling pathway, and adrenergic signaling in cardiomyocytes. We speculated that DEGs may influence the occurrence and progression of IS through these potential pathways. Furthermore, GSEA and GSVA results showed that IS was influenced by the inflammation and immune regulation pathway, which is consistent with the current theory that inflammation and immune responses play key roles in the regulatory network of IS (Endres et al., 2022).

In this study, we constructed a diagnostic model of IS and identified four characteristic genes (*GFAP*, *GPNMB*, *FKBP9*, and *CHMP5*) with good diagnostic value. The results of RT-qPCR showed that these four characteristic genes were significantly upregulated in a mouse MCAO model, which verified our results. Glial fibrillary acidic protein (GFAP), an intermediate filament protein only produced by astrocytes, is a well-established marker of astrocyte activation in central nervous system (CNS) diseases (Sayad et al., 2022). Growing evidence suggests the potential clinical application value of blood GFAP levels in numerous neuroinflammatory and neurodegenerative diseases, as they can be used to detect even subtle injury to the CNS (Abdelhak et al., 2022; Heimfarth et al., 2022). A previous study has shown that IS can induce the transformation of astrocytes into a neurotoxic A1 phenotype and increase GFAP expression (Zhang et al., 2022). Amalia, 2021 reported that GFAP is highly expressed in cerebrospinal fluid and serum from patients with IS, demonstrating its potential as a reliable biological marker to help diagnose IS. Our results revealed a similar expression tendency of GFAP as these reports.

Glycoprotein nonmetastatic melanoma protein B (GPNMB) is a type-I transmembrane protein, also known as dendritic cell heparan sulfate proteoglycan integrin-dependent ligand, that has been demonstrated to be overexpressed in numerous cancers and is associated with a metastatic phenotype (Huang et al., 2021). It has been reported that tumor endothelial cells can induce tumor-infiltrating CD8 T cell exhaustion and promote the escape of cancer cells from immune surveillance by upregulating the expression of GPNMB (Sakano et al., 2022). Furthermore, GPNMB plays an important role in various diseases in addition to cancer by regulating inflammation and immune responses. GPNMB can negatively regulate macrophage inflammatory capacity *via* the inhibition of NF-κB signaling by binding to CD44 (Prabata et al., 2021). In a cellular amyotrophic lateral sclerosis model, it has been shown that GPNMB exerts neuroprotective effects by binding to Na/K-ATPase, an ion pump and receptor that modulates neuroinflammation (Ono et al., 2016). Consistent with our results, Nakano et al. (2014) have demonstrated that GPNMB is upregulated after ischemic reperfusion, and the overexpression of GPNMB has neuroprotective effects against IS, although the mechanism has not been fully characterized.

FK506-binding protein 9 (FKBP9), a member of the immunophilin family FKBPs, binds to the immunosuppressive drug tacrolimus (FK506) (Ghartey-Kwansah et al., 2018). FKBP9 is widely expressed in multiple human organs and tissues and involved in the regulation of various physiological processes. It has been reported that FKBP9 is associated with metastasis and poor prognosis in a variety of cancers (Annett et al., 2020). For example, Xu H. et al. (2020) demonstrated that FKBP9 is upregulated in human glioblastoma samples and promotes malignant phenotypes by regulating unfolded protein response signaling. Additionally, FKBP9 is closely related to physiological functions such as T cell activation and plays an important role in immune system regulation (Jiang et al., 2020).

Charged multivesicular body protein 5 (CHMP5), a component of the endosomal sorting complex required for transport-III, is responsible for the final conversion of late endosomal multivesicular body to lysosomes (Shim et al., 2006). CHMP5 is a multifunctional protein with potential roles in cellular signaling. It was previously reported that CHMP5 has antiapoptotic functions because silencing CHMP5 induces apoptosis by caspase cascade activation (Mo et al., 2018). Additionally, CHMP5 prevents Bcl-2, a widely recognized apoptosis suppressor gene that intrinsically regulates apoptosis, from deleterious oxidation by reactive oxygen species (ROS) formation (Adoro et al., 2017). Furthermore, it has been shown that CHMP5 has a key role in T-cell receptor signaling and its deficiency affects T-cell receptor expression on the cell surface (Wi et al., 2016). However, the role of CHMP5 in IS has not been studied to date.

In terms of the immune response, the infiltration of Tregs was significantly lower in IS samples than in control samples. This result is consistent with the previous findings of Noh et al. (2018), indicating that Tregs are closely related to the pathogenesis of IS. Tregs are an important subpopulation of T lymphocytes that are involved in resisting immune response overactivity, maintaining immune homeostasis, and regulating inflammation (Wang et al., 2021). In Figure 3, immune infiltration levels were evaluated through Cibersort algorithm. Total eight types of immune cells were found to be of statistical significance, Treg cells were the most significant among them. In Figure 6, Several species of immune cells including Eosinophils, Treg and so on were of significant correlation relationship (|Correlation coefficient| > 0.5) with four characteristic genes. Treg, being of the most significant statistical meanings between IS and control, were closely negatively related with all characteristic genes. Activation of Treg has been verified to slow down the process of progress of IR through reducing IFN-γin the IR microenvironment (Hu et al., 2013). GPNMB protein could bind to heparan sulphate-like structures, blocking the activation of T cells (Chung et al., 2013). These are consistent with our results.

Our study showed that the four IS RNA methylation-related characteristic genes were significantly negatively correlated with the degree of Tregs infiltration, indicating that these genes participate in the immune regulation of IS. M0 macrophage is another important cell type in immune infiltration, was found to secret interleukin-1βaccompanying with the progress of early IR (Mabuchi et al., 2000). Thus, accumulation of interleukin-1βcan promote the development of follicular helper T cells (Kobayashi et al., 2017), been seen as a bridge linking M0 macrophages with follicular helper T cells in our study.

We built a PPI network and identified *GPNMB* as a hub gene. miRNAs are a class of small, single-stranded noncoding RNAs that regulate target gene expression on a post-transcriptional level (Correia de Sousa et al., 2019). In our study, miR-26b-5p was predicted to act on GPNMB, and the expression of GPNMB in IS samples was upregulated. Previous studies have shown that miR-26b-5p is associated with various disease states, such as tumors, inflammation, autoimmune disease, and IS. For example, a bioinformatics analysis reported that miR-26b-5p can be recognized as a potential biomarker for IS (Barrera-Vázquez et al., 2022). Additionally, Xiao et al. (2021) have observed that miR-26b-5p is downregulated in the brain of an MCAO rat model and that the overexpression of miR-26b-5p reduces apoptosis and the inflammatory response. Another study has shown that miR-26b-5p alleviates IS injury by negatively regulating the expression of Smad1, which promotes apoptosis and inflammation by increasing the level of ROS in cells (Shangguan et al., 2020). The findings of these reports are consistent with our results.

Finally, we predicted potential drug molecules that may bind to GPNMB, and the most prominent was chloroquine. Chloroquine, an established drug originally used for the treatment of malaria, has been reported to have anti-inflammatory and immunomodulatory properties (Silva et al., 2021). Recently, several studies have shown that chloroquine pretreatment can alleviate brain injury in IS through a variety of mechanisms, including the inhibition of the inflammatory response by lowering myeloperoxidase activity and inflammatory cytokine gene expression (Cui et al., 2013; Zhang Y. P. et al., 2020) and alleviation of neuronal injury by restoring ganglioside homeostasis (Caughlin et al., 2019). Gabriel et al. (2014) reported that chloroquine can effectively increase *Gpnmb* transcription in mice as a lysosomal stress inducer. All these reports are mostly consistent with our analysis.

There were some limitations to this study. First, single microarray analysis may be associated with high false-positive rates, and it is necessary to integrate multiple individual datasets in future studies to improve the reliability of the results. Second, although the clinical diagnostic model constructed in this study showed high accuracy, the sample sizes of the training and validation sets were small, resulting in insufficient statistical efficacy. Performing cross-validation internally and increasing the sample size for external validation in future studies would be beneficial. Finally, our research was retrospective, and a large number of prospective studies are needed to validate the results.

In conclusion, this study is the first to comprehensively analyze the correlations between RNA methylation-related regulators and IS and immune infiltration. We identified two highly heterogenous RNA methylation subtypes in IS, with significantly different BP and MF. An IS clinical diagnosis model was constructed and four characteristic genes with effective diagnostic value were identified using bioinformatics methodologies (such as WGCNA and LASSO regression). *GPNMB* was identified as a hub gene by PPI network analysis, and its regulatory networks and binding to the potential therapeutic drug chloroquine may provide guidance for clinical diagnosis and treatment. Overall, our study may provide insight into the potential molecular mechanisms underlying IS and a new basis for optimizing the clinical diagnosis and treatment of patients with IS.

## Data Availability

The datasets presented in this study can be found in online repositories. The names of the repository/repositories and accession number(s) can be found in the article/Supplementary Material.
