# Supplementary figures and images for: Expression pattern and clinical value of Key RNA methylation modification regulators in ischemic stroke
Source: Front Genet. 2022 Oct 3;13:1009145. doi: 10.3389/fgene.2022.1009145 (PMC9574037; doi:10.3389/fgene.2022.1009145)

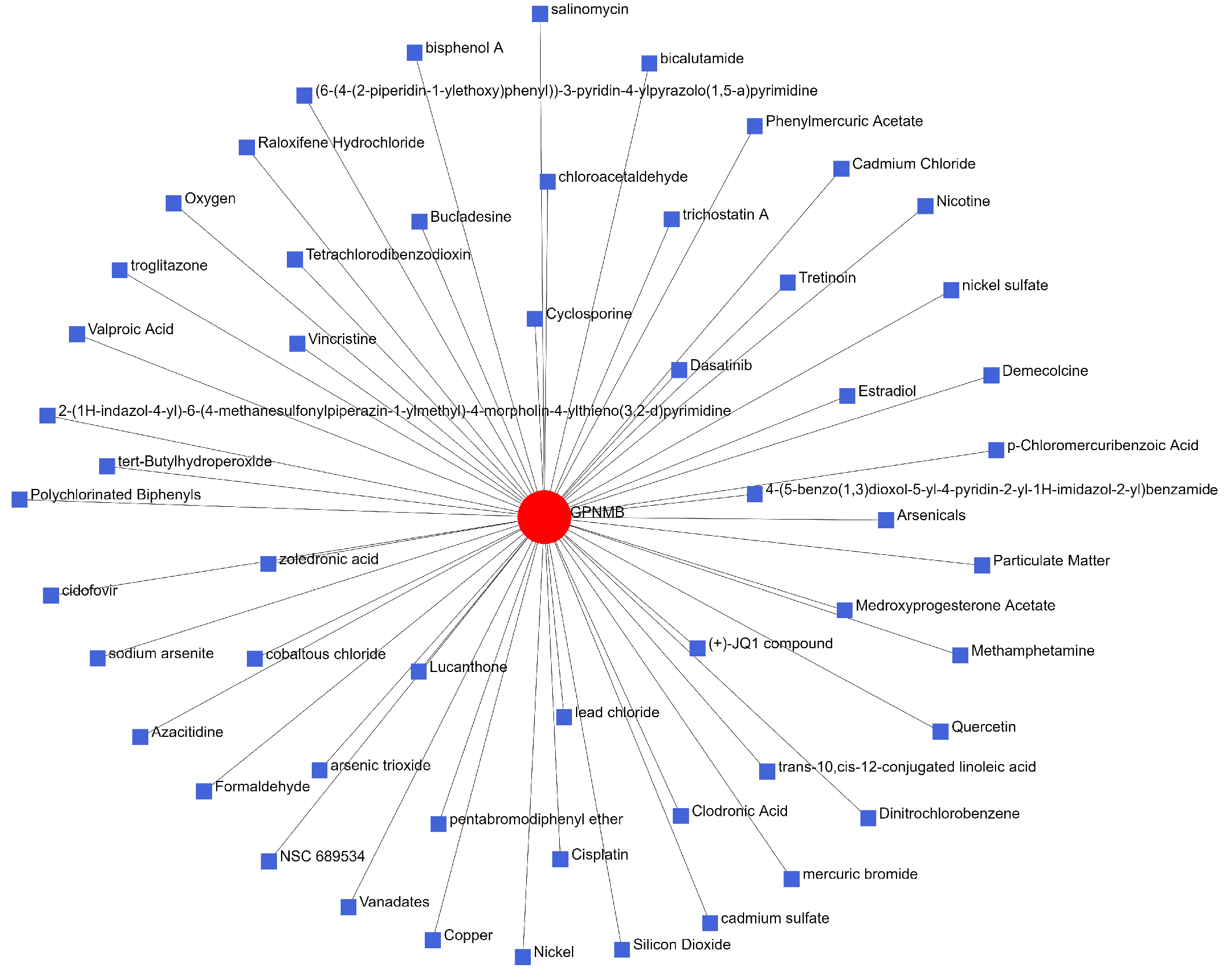

Supplement: Supplementary file 2 [file Image6.TIF]

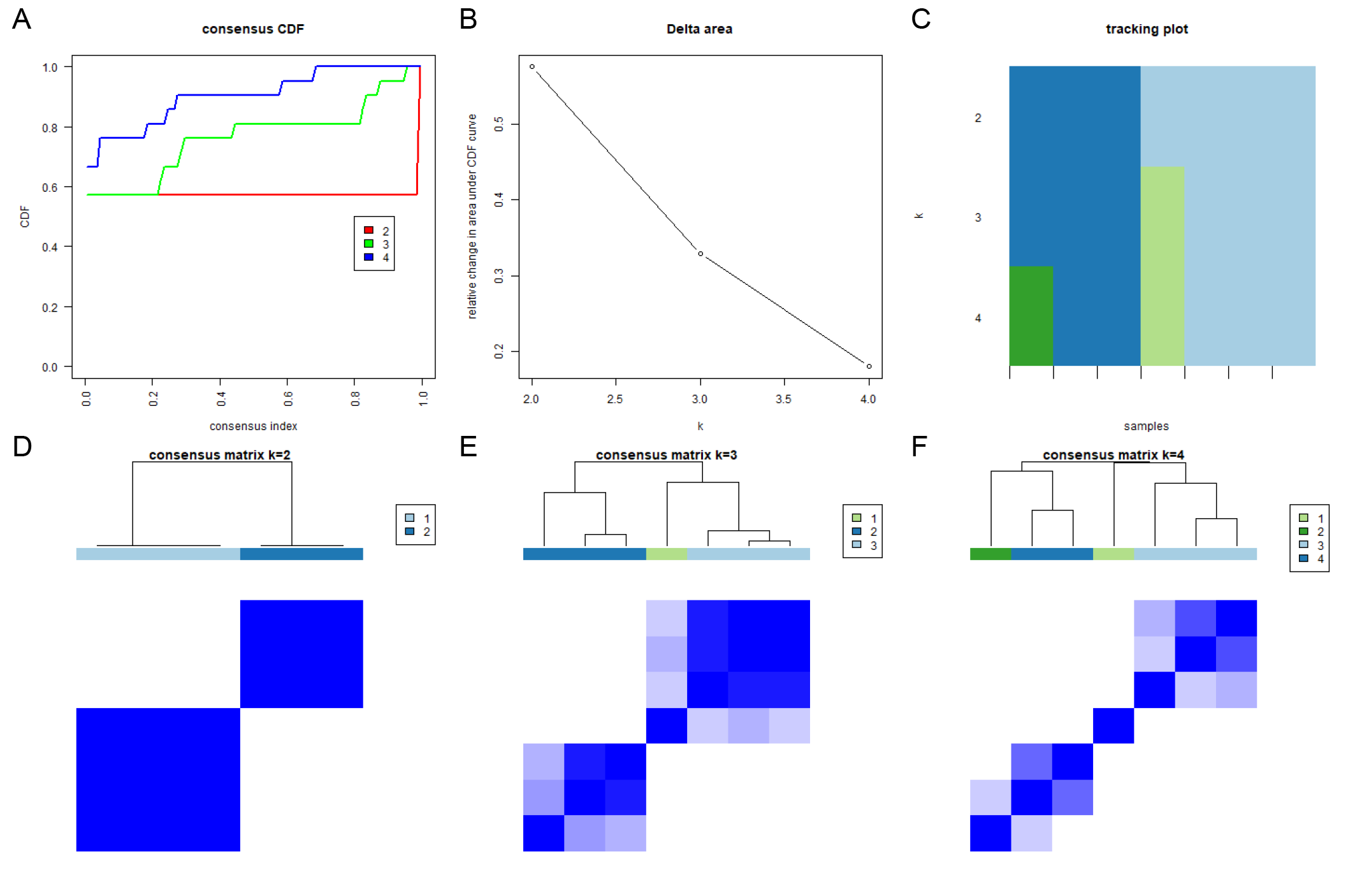

Supplement: Supplementary file 3 [file Image3.TIF]

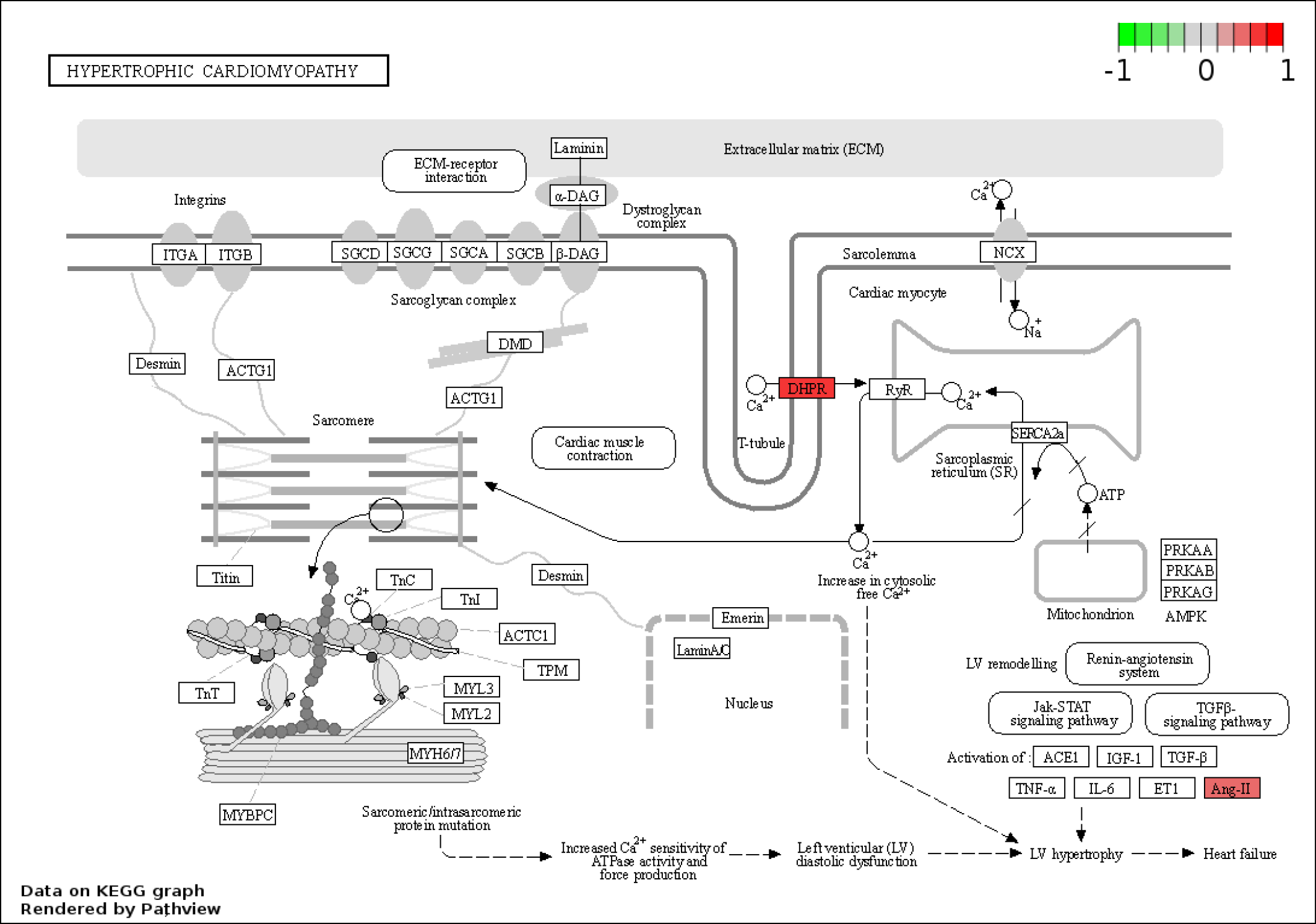

Supplement: Supplementary file 4 [file Image4.TIF]

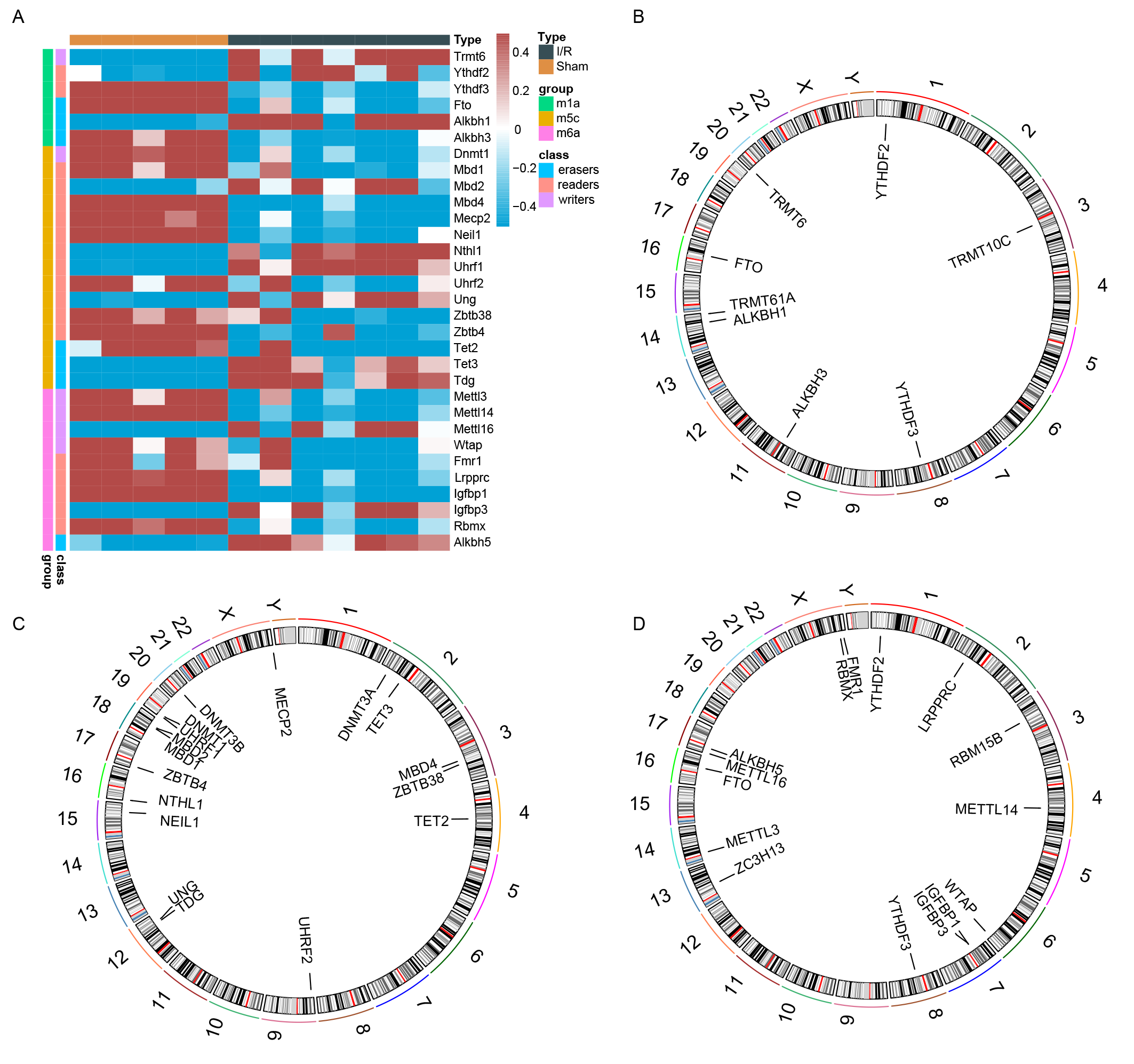

Supplement: Supplementary file 5 [file Image2.TIF]

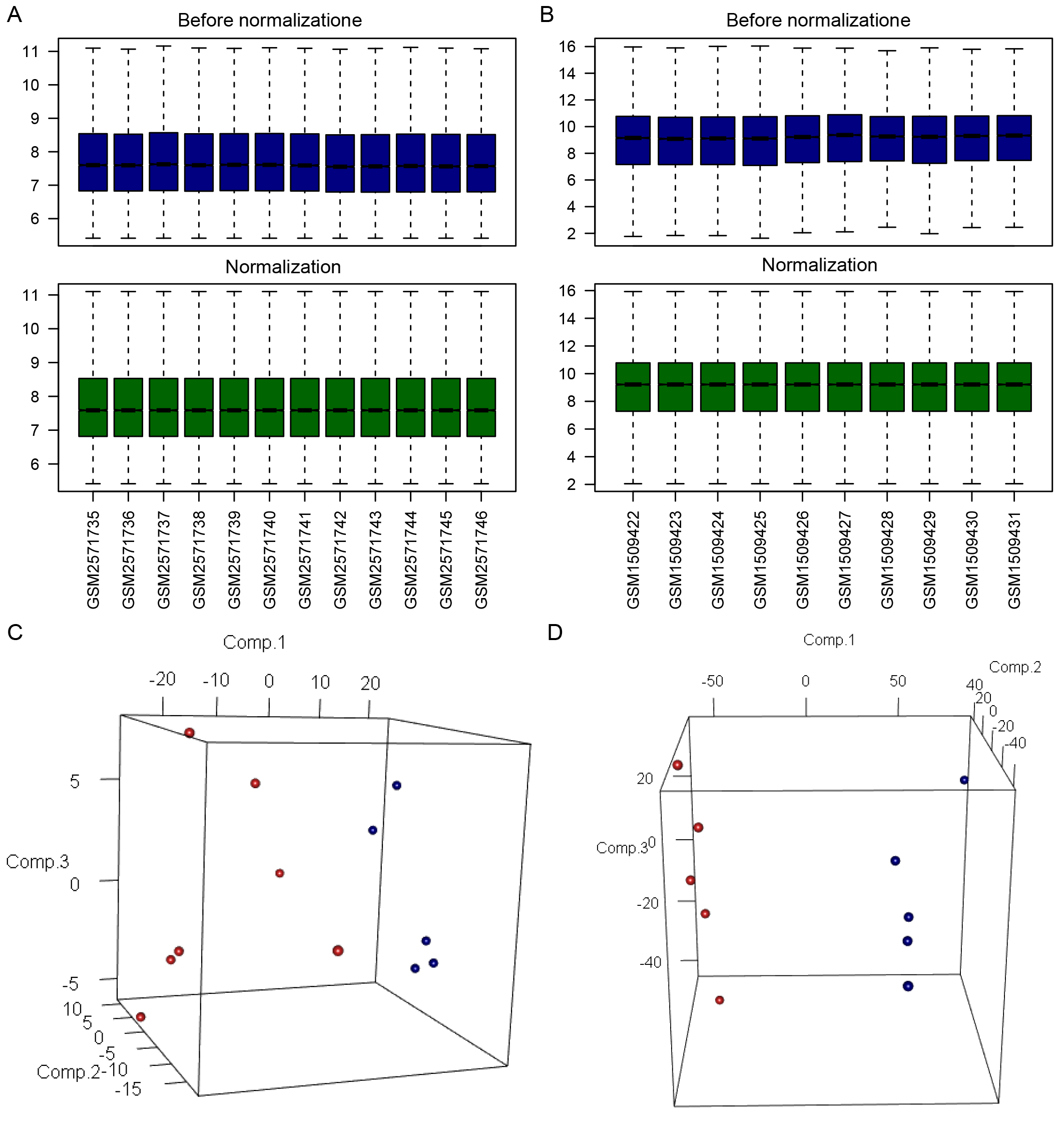

Supplement: Supplementary file 6 [file Image1.TIF]

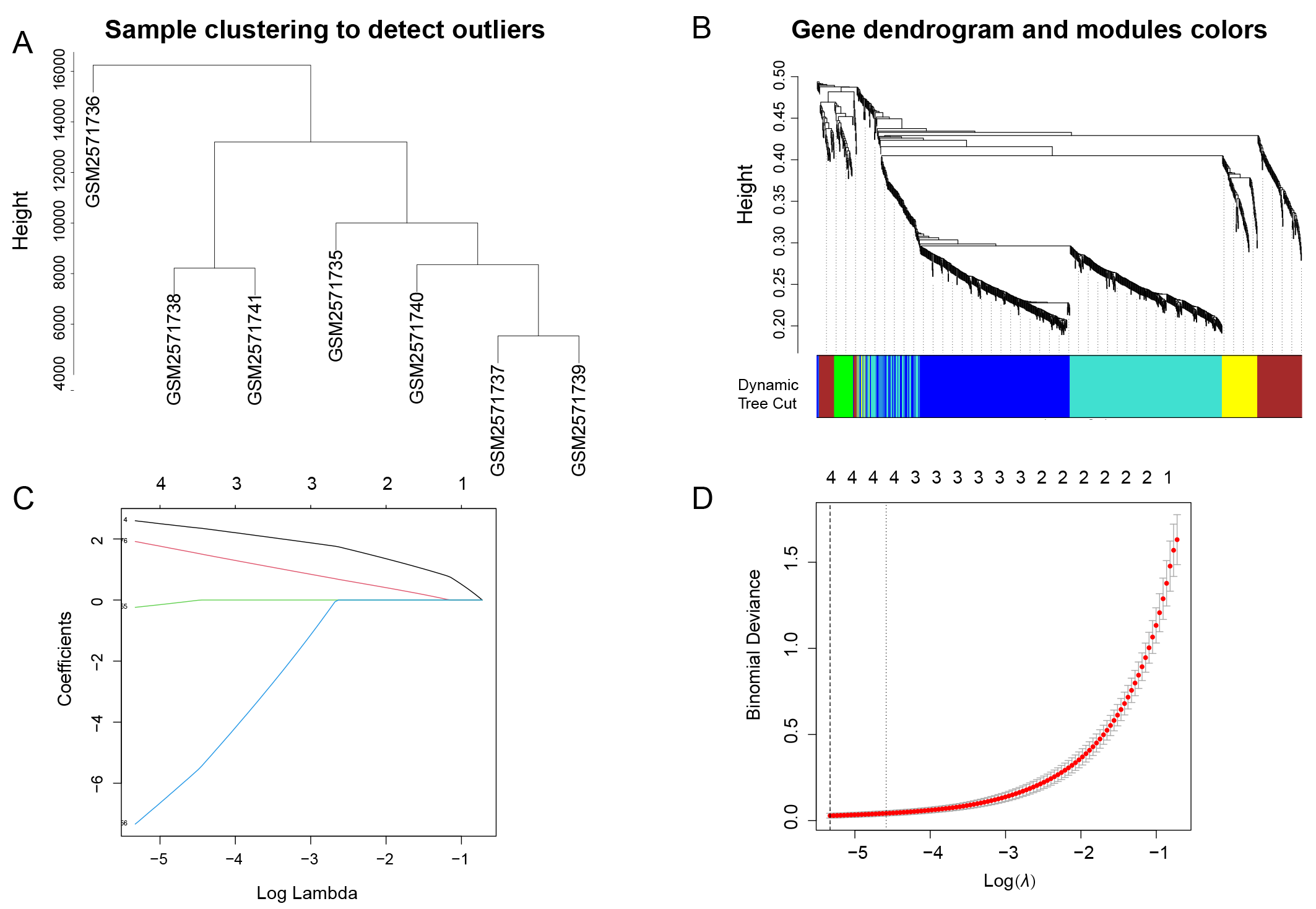

Supplement: Supplementary file 9 [file Image5.TIF]
