# Supplementary material for: Expression pattern and clinical value of Key RNA methylation modification regulators in ischemic stroke
Source: Front Genet. 2022 Oct 3;13:1009145. doi: 10.3389/fgene.2022.1009145 (PMC9574037; doi:10.3389/fgene.2022.1009145)
Supplement: Supplementary file 7 [file Table3.DOCX]

Supplementary Material

# Supplementary Figures and Tables

## Supplementary Tables

**Supplementary Table S1**. The detailed information of GSE97537 and GSE61616

|  | **GSE97537** | **GSE61616** |
| --- | --- | --- |
| **Organism** | Rattus norvegicus | Rattus norvegicus |
| **Tissue** | Cerebral | Cerebral |
| **Experiment type** | profiling by array | profiling by array |
| **Platforms** | GPL1355 | GPL1355 |
| **Gene (number)** | 14373 | 14373 |
| **Sample (number)** |  |  |
| Control/Sham | 5 | 5 |
| Cerebral I/R | 7 | 5 |
| Total | 12 | 10 |

**Supplementary Table S2**. The primer sequence information of PCR experiment

| Targets | Primers | Sequence (5' to 3') | Product length (bp) | |
| --- | --- | --- | --- | --- |
| GFAP | Forward | GCGAAGAAAACCGCATCACC | 180 |  |
|  | Reverse | AAGGGAGAGCTGGCAGG |  |  |
| GPNMB | Forward | CTATCCCTGGCAAAGACCCA | 221 |  |
|  | Reverse | CTGGTCTCCTCGGAAGAACG |  |  |
| FKBP9 | Forward | GAGCTTCGTGCCTGATGAGT | 116 |  |
|  | Reverse | AGTCCCTGTCATAGCTGGAGT |  |  |
| CHMP5 | Forward | CGGAAAAGCGAAACCCAAGG | 221 |  |
|  | Reverse | GCTGTTGCTCATACATCCGC |  |  |

## Supplementary Figures


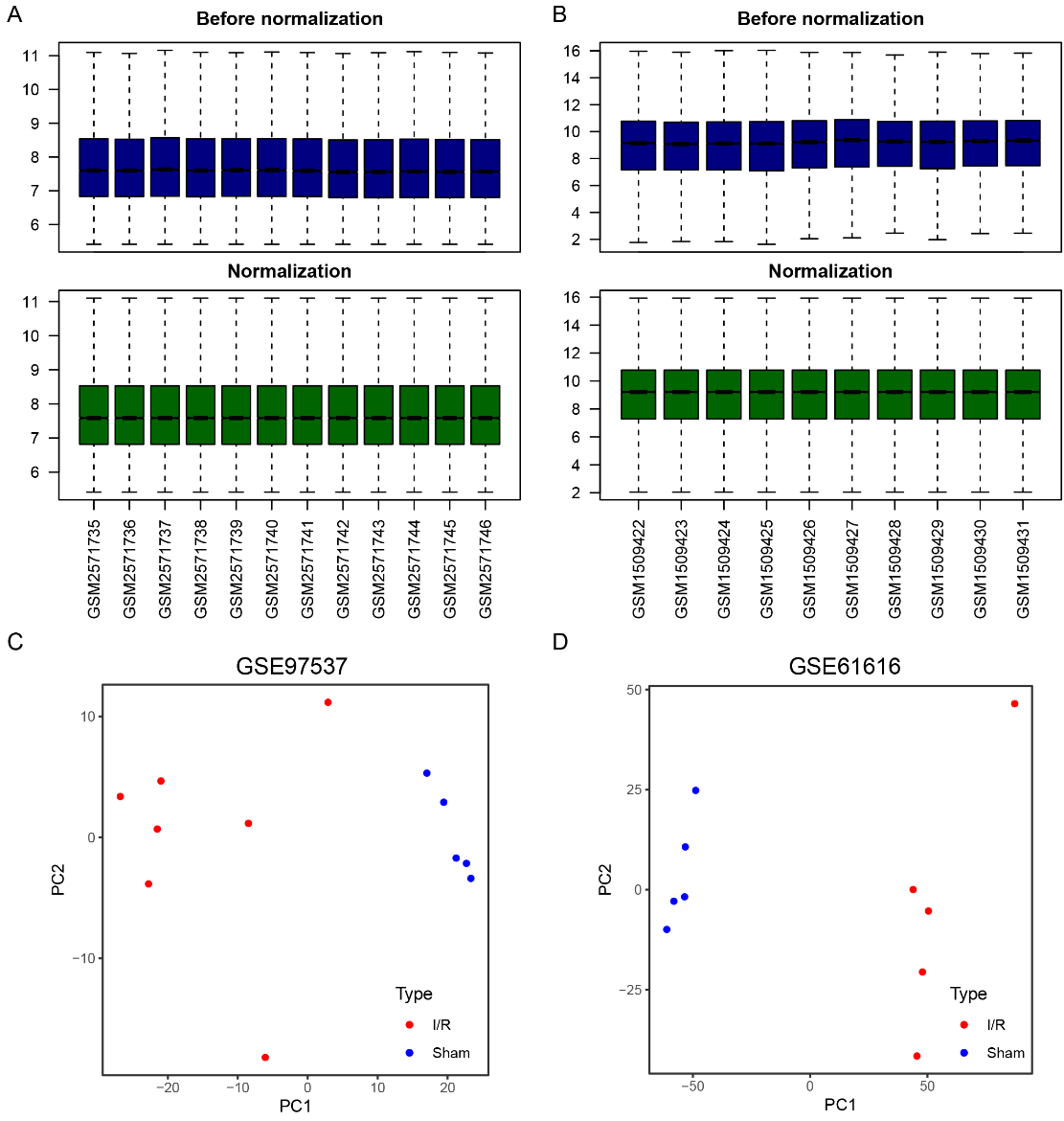


**Supplementary Figure 1.** Data preprocessing. (A) GSE97537 dataset before and after normalization. (B) GSE61616 dataset before and after normalization. Sample distributions of the ischemic stroke (IS) and control groups in GSE97537 dataset (C) and GSE61616 dataset (D); blue represents control group and red represents the IS group.


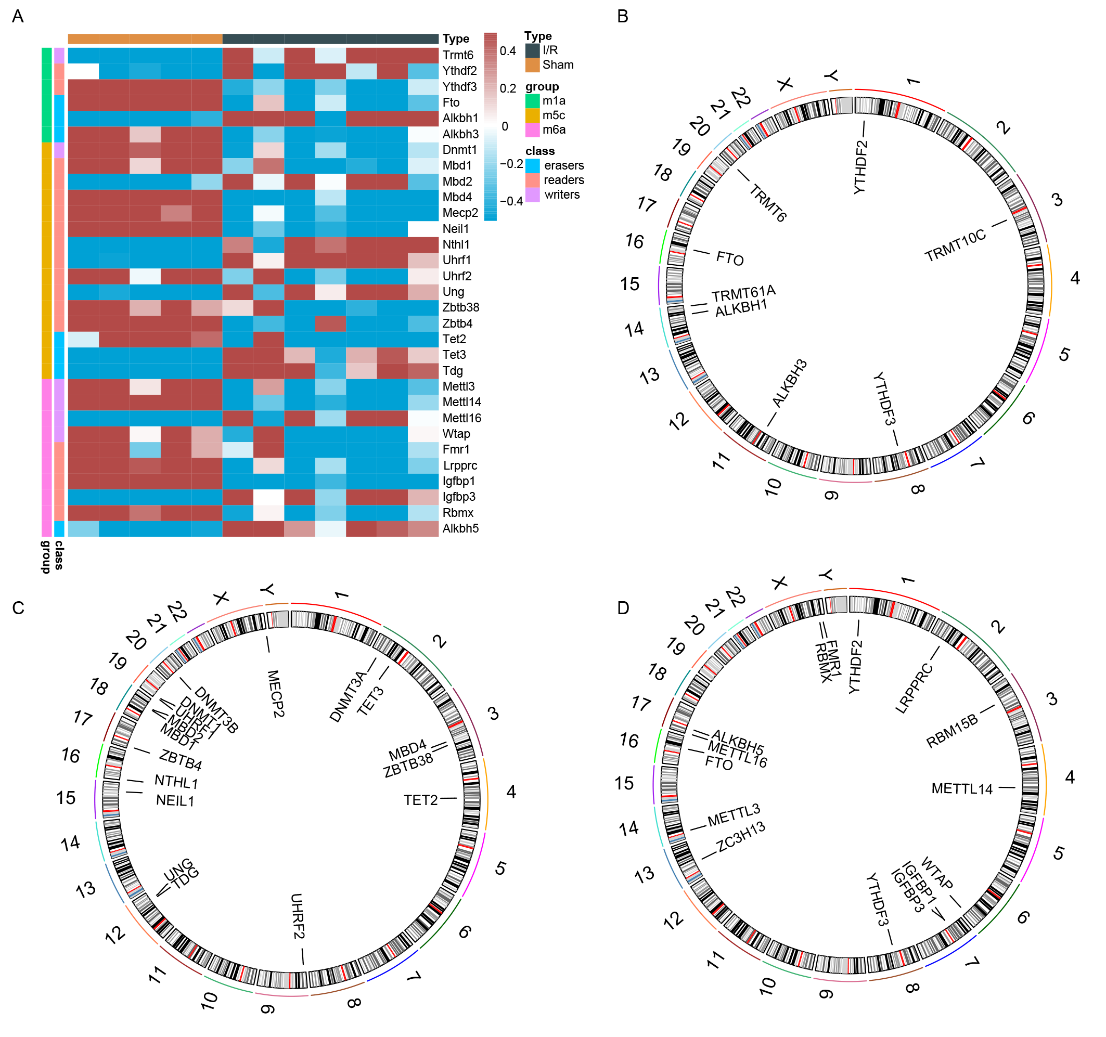


**Supplementary Figure 2.** Differentially expressed genes (DEGs) and chromosomal localizations. (A) Heat map of part of the DEGs between ischemic stroke and control groups. Red indicates upregulated and blue represents downregulated expression levels. (B–D) Chromosomal localization. Chromosome localization map of differentially expressed m1A regulators (B), m5C regulators (C), and m6A regulators (D).


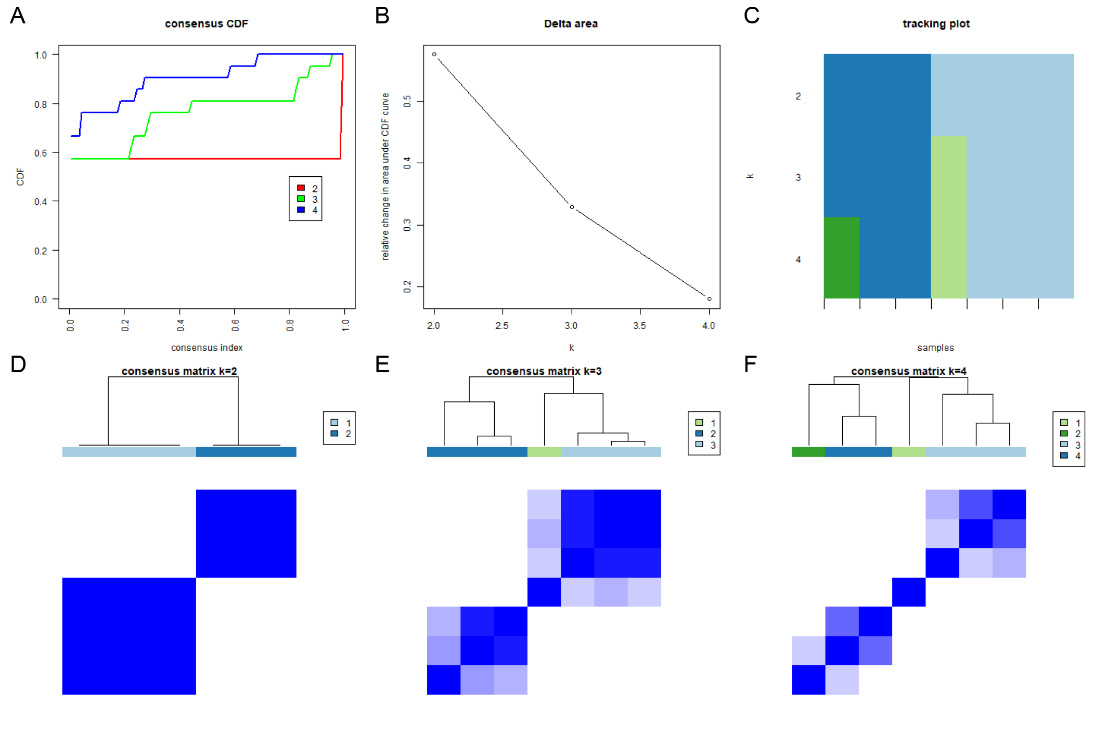


**Supplementary Figure 3.** Identifying ischemic stroke (IS) molecular subtypes based on the results of cluster analysis. (A) Consensus clustering cumulative distribution function (CDF) plots; the abscissa is the consensus index and the ordinate is the CDF index. (B) Delta area plots. (C) Tracking plot. (D–F) Cluster heat map of IS molecular subtypes when k = 2 (D), k = 3 (E), and k = 4 (F).


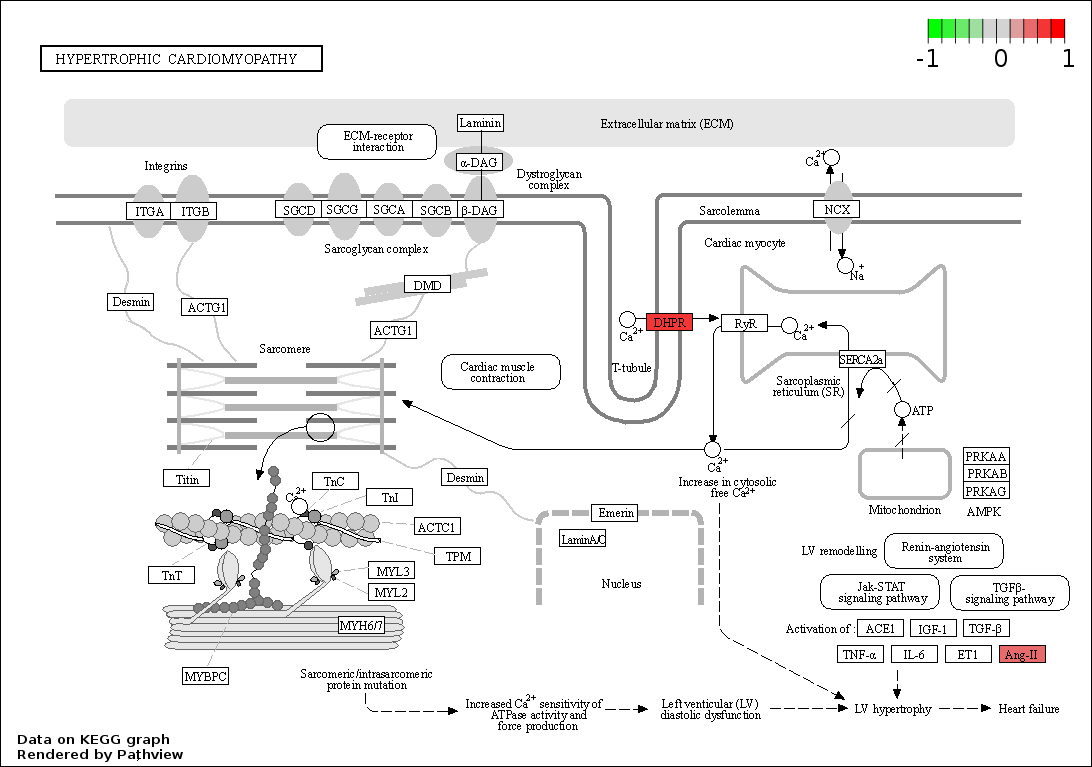


**Supplementary Figure 4.** Significantly enriched KEGG pathway: Hypertrophic cardiomyopathy.


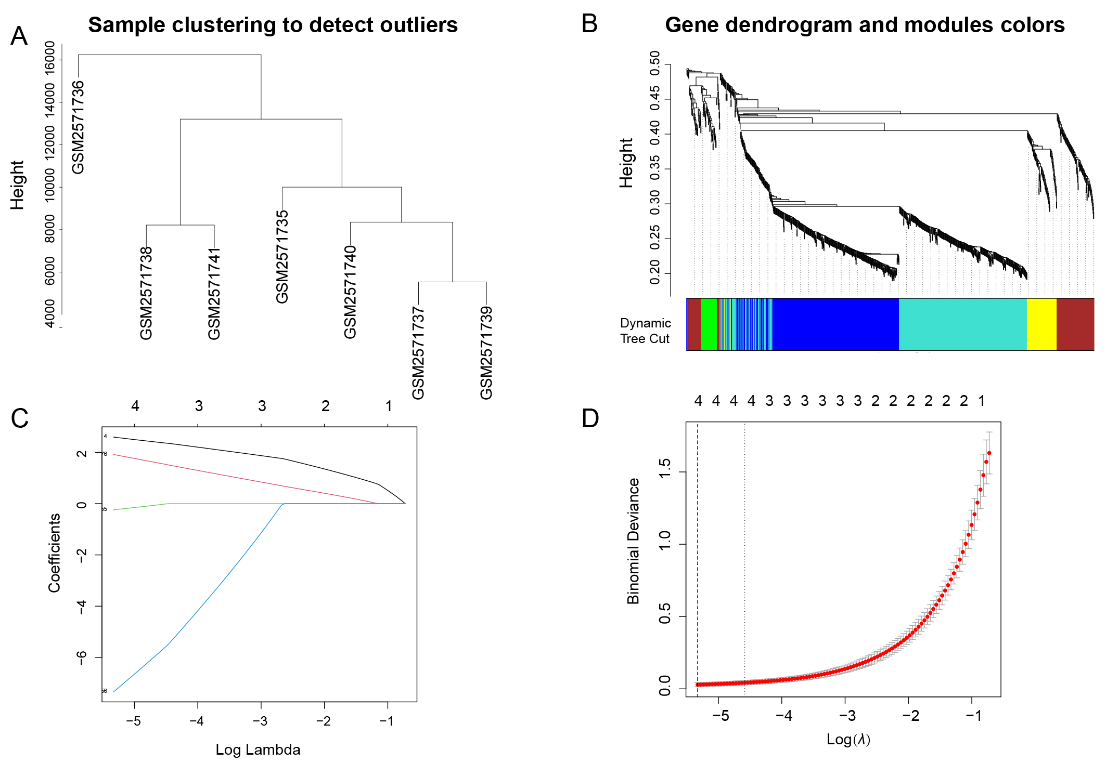


**Supplementary Figure 5.** Weighted gene co-expression network analysis and the construction of diagnostic model. (A) No outlier sample was detected in the sample clustering procedure. (B) Gene dendrogram and module colors. (C) Relationship between the selected characteristic parameters and absolute value of coefficients. (D) Optimal model and minimalist model were obtained.

**
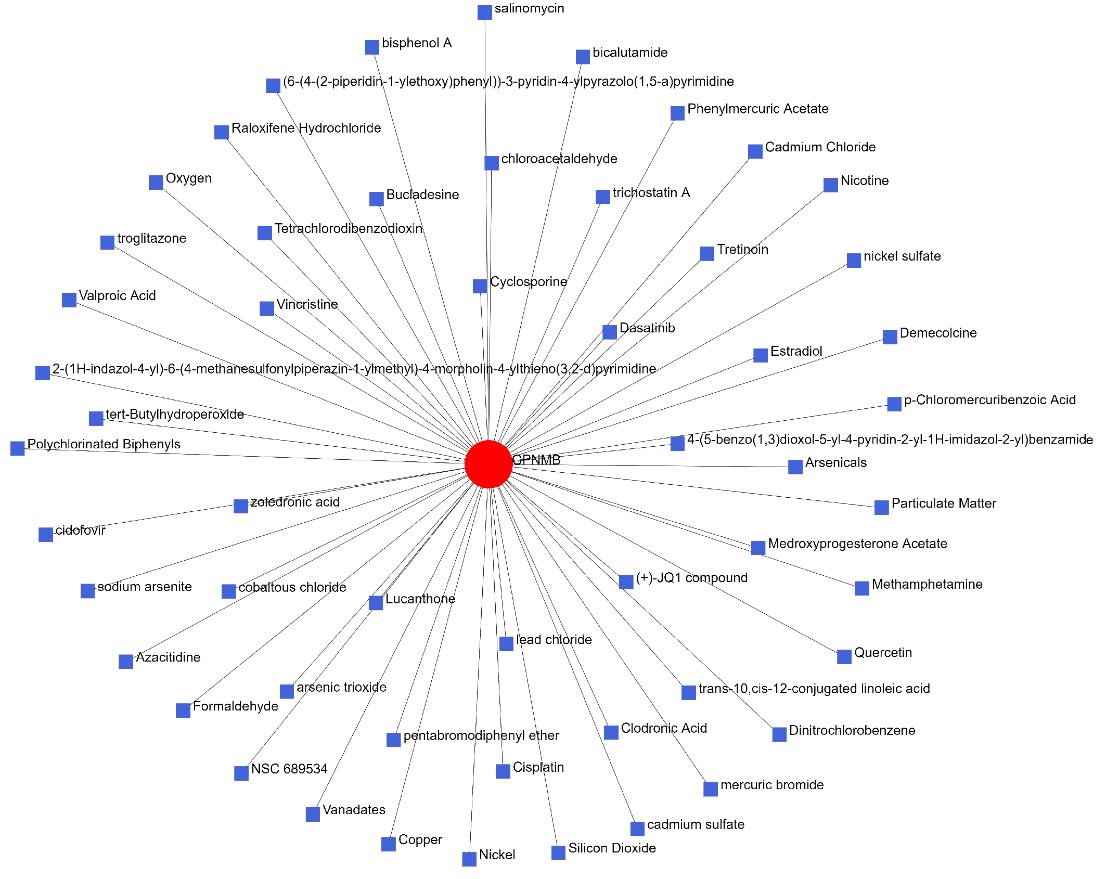
**

**Supplementary Figure 6.** GPNMB-drug interaction networks.
